# Crystal structures of REF6 and its complex with DNA reveal diverse recognition mechanisms

**DOI:** 10.1038/s41421-020-0150-6

**Published:** 2020-03-31

**Authors:** Zizi Tian, Xiaorong Li, Min Li, Wei Wu, Manfeng Zhang, Chenjun Tang, Zhihui Li, Yunlong Liu, Zhenhang Chen, Meiting Yang, Lulu Ma, Cody Caba, Yufeng Tong, Hon-Ming Lam, Shaodong Dai, Zhongzhou Chen

**Affiliations:** 1 State Key Laboratory of Agrobiotechnology and Beijing Advanced Innovation Center for Food Nutrition and Human Health, College of Biological Sciences, China Agricultural University, 100193 Beijing, China; 2 Department of Chemistry and Biochemistry, University of Windsor, Windsor, ON N9B 3P4 Canada; 3 School of Life Sciences and Center for Soybean Research of the State Key Laboratory of Agrobiotechnology, The Chinese University of Hong Kong, Shatin, N.T., Hong Kong SAR, China; 4 Department of Pharmaceutical Sciences, Skaggs School of Pharmacy and Pharmaceutical Sciences, University of Colorado Anschutz Medical Campus, Aurora, CO 80045 USA

**Keywords:** Methylation, Plant molecular biology, X-ray crystallography

## Abstract

Relative of Early Flowing 6 (REF6) is a DNA-sequence-specific H3K27me3/2 demethylase that contains four zinc finger (ZnF) domains and targets several thousand genes in *Arabidopsis thaliana*. The ZnF domains are essential for binding target genes, but the structural basis remains unclear. Here, we determined crystal structures of the ZnF domains and REF6-DNA complex, revealing a unique REF6-family-specific half-cross-braced ZnF (RCZ) domain and two C2H2-type ZnFs. DNA-binding induces a profound conformational change in the hinge region of REF6. Each REF6 recognizes six bases and DNA methylation reduces the binding affinity. Both the acidic region and basic region are important for the self-association of REF6. The REF6 DNA-binding affinity is determined by the sequence-dependent conformations of DNA and also the cooperativity in different target motifs. The conformational plasticity enables REF6 to function as a global transcriptional regulator that directly binds to many diverse genes, revealing the structural basis for the epigenetic modification recognition.

## Introduction

Histones are subjected to comprehensive post-translational modifications, including methylation, acetylation, phosphorylation, and ubiquitination. Histone methylation generally occurs on lysine and arginine residues of the N-terminal tails and this can provide an activating or repressive epigenetic mark. H3K27 methylation plays a key role in gene repression and developmental regulation. Most histone demethylases are JmjC-domain-containing proteins and are highly conserved^1–4^. However, no homologs of metazoan H3K27-specific demethylases KDM6a or KDM6b were found in *Arabidopsis*^5^. Both Relative of Early Flowering 6 (REF6)^6^ and Early Flowering 6 (ELF6)^7^ have been shown to be specific H3K27me3/2 demethylases in plants. JMJ13 is also an H3K27me3 site-specific demethylase, but has no significant activity on H3K27me2^8^. REF6, ELF6 and JMJ13 are homologous and belong to the KDM4 subfamily. REF6 and ELF6 share a high sequence similarity comprising an N-terminal Jumonji N (JmjN) domain, a JmjC domain, and tandem zinc finger (ZnF) domains in the C-terminus. However, these two demethylases have divergent roles in the regulation of flowering time^9,10^. Mutants of *elf6* display an earlier floral transition compared with wild type, whereas mutations in *ref6* cause later flowering under different lighting conditions^9^.

Classical C2H2 ZnF domains comprise the largest class of DNA-binding domains^11^ and are related with various biological processes, including recombination, development, and chromatin regulation. Many histone demethylases contain specialized ZnF domains, such as PHD, Zn-CW, and RING^1,5^ that are involved in histones binding or ubiquitination^12^, as well as other functions. However, C2H2 Zinc fingers, which are well established as DNA binding modules, are rarely found in histone demethylases.

The ZnFs of REF6 are found to bind CTCTGYTY (where Y is C or T) DNA motifs directly and recognize specific nucleosomes^13,14^. Although REF6 retains its enzymatic activity in the absence of the four ZnF domains in vitro, it fails to compensate the late-flowering and short-petiole phenotypes of the *ref6* mutant in *Arabidopsis*^13,14^. Therefore, the ZnF domains are indispensable for REF6 function in vivo. Furthermore, REF6 dysfunction increases the levels of H3K27me3 and suppresses the expression of hundreds of endogenous genes, resulting in various phenotypic defects^6^. Sequence alignment shows that the ZnF domains are conserved among different plant species. These results indicate that the ZnF domains are essential for the binding to DNA in a sequence-specific manner. These observations also identify a new targeting mechanism for the recruitment of histone demethylases directly by specific DNA sequences.

A total of 2836 genes are reported to be regulated by REF6^13^. Interestingly, not all REF6-bound DNAs contain the CTCTGYTY motif. About 80% of REF6-targeted DNAs contain the motif and only 15% of the CTCTGYTY motifs in the *Arabidopsis* genome are recognized by REF6^13,14^. This indicates that the sequence is not the only requirement of the REF6−DNA interaction. The structural basis of how REF6 recognizes and binds diverse DNA motifs lacks understanding, yet this information is vital to reveal its role as a transcriptional regulator.

In this study, we present crystal structures of the ZnF domains of REF6 and its complex with dsDNA. We identified a novel REF6-family-specific half-cross-braced ZnF (RCZ) domain, a hinge, an acidic region (residues 1224–1239), a basic region (residues 1355–1360), and two classical C2H2 ZnF domains in the structure. Although each REF6 binds directly to six bases, the binding ability and specificity are markedly enhanced by a combination of DNA base and shape readout, and the cooperativity of the binding motifs. Our results reveal a novel mechanism of how the conformational plasticity of DNA enables REF6 to recognize diverse target genes.

## Results

### REF6 contains an uncommon half-cross-braced ZnF (RCZ) domain

**Fig. 1:**
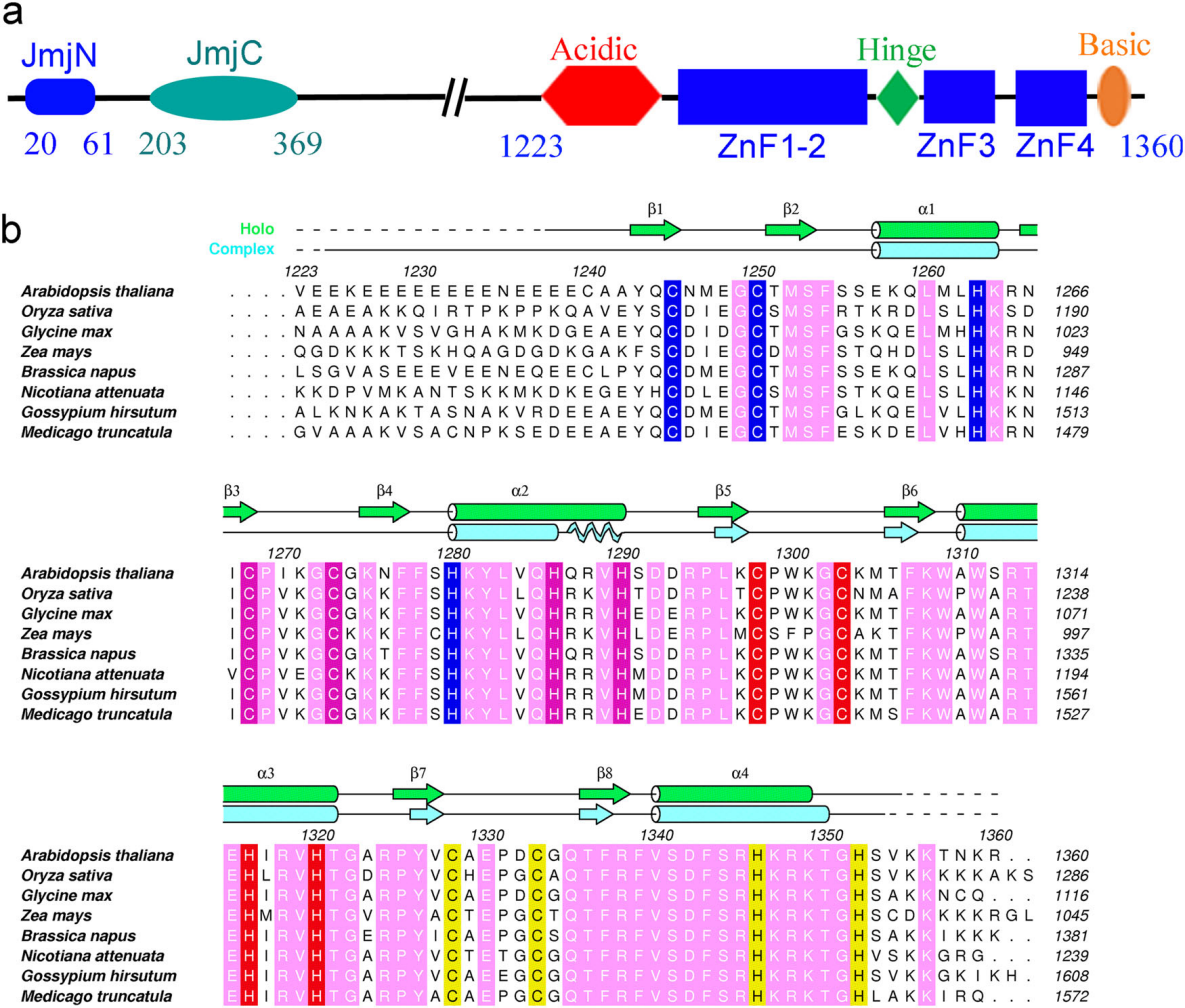
Overview of the REF6 domains. **a** Domain architecture of the REF6 protein based on the solved structure. Domain boundaries are indicated by residue numbers. JmjN, JmjC, acidic region, ZnF1-2 domain, hinge, ZnF3 domain, ZnF4 domain, and basic region are shown. **b** Sequence alignment of REF6 proteins from different species. Secondary structural elements of the DNA-free and DNA-bound REF6 structure are calculated using DSSP and colored in green and cyan, respectively. Cylinders, waved lines and arrows represent α-helices, 3_10_ helices, and β-strands, respectively. The residues coordinating the four Zn^2+^ ions are shown as blue, magenta, red, and yellow, respectively. The invariant residues between different species are shown in pink. The sequences used are: REF6 from *Arabidopsis thaliana* (NP_680116.2), lysine-specific demethylase JMJ705 from *Oryza sativa Japonica Group* (XP_015621377.1), GLYMA_04G192000 from *Glycine max* (KRH63699.1), REF6 from *Zea mays* (AQK40026.1), REF6 from *Brassica napus* (XP_013724852.1), REF6 from *Nicotiana attenuata* (XP_019243504.1), REF6 from *Gossypium hirsutum* (XP_016718821.1), REF6 from *Medicago truncatula* (XP_013460954.1).

To dissect the structure−function relationship of functionally important domains of REF6, we attempted to express and purify a series of truncated REF6 fragments in *E. coli*. After extensive trials, only one fragment of REF6 (residues 1223–1360) that contains all four zinc fingers (Fig. 1a, b) and is capable of binding the CTCTGYTY motifs produced high-quality crystals at 18 °C. The number and initial positions of the zinc ions (Supplementary Fig. S1) were then determined using the single-wavelength anomalous diffraction (SAD) method by the SHELX C/D/E program^15^. The final holo-REF6 structure in the space group *P*4_1_ was refined to 1.57 Å resolution (Supplementary Table S1). The structure consists of residues 1238–1353, lacking the N-terminal acidic region and the C-terminal basic region. This is mainly due to conformational flexibility rather than protein degradation during crystallization (Supplementary Fig. S2).

**Fig. 2:**
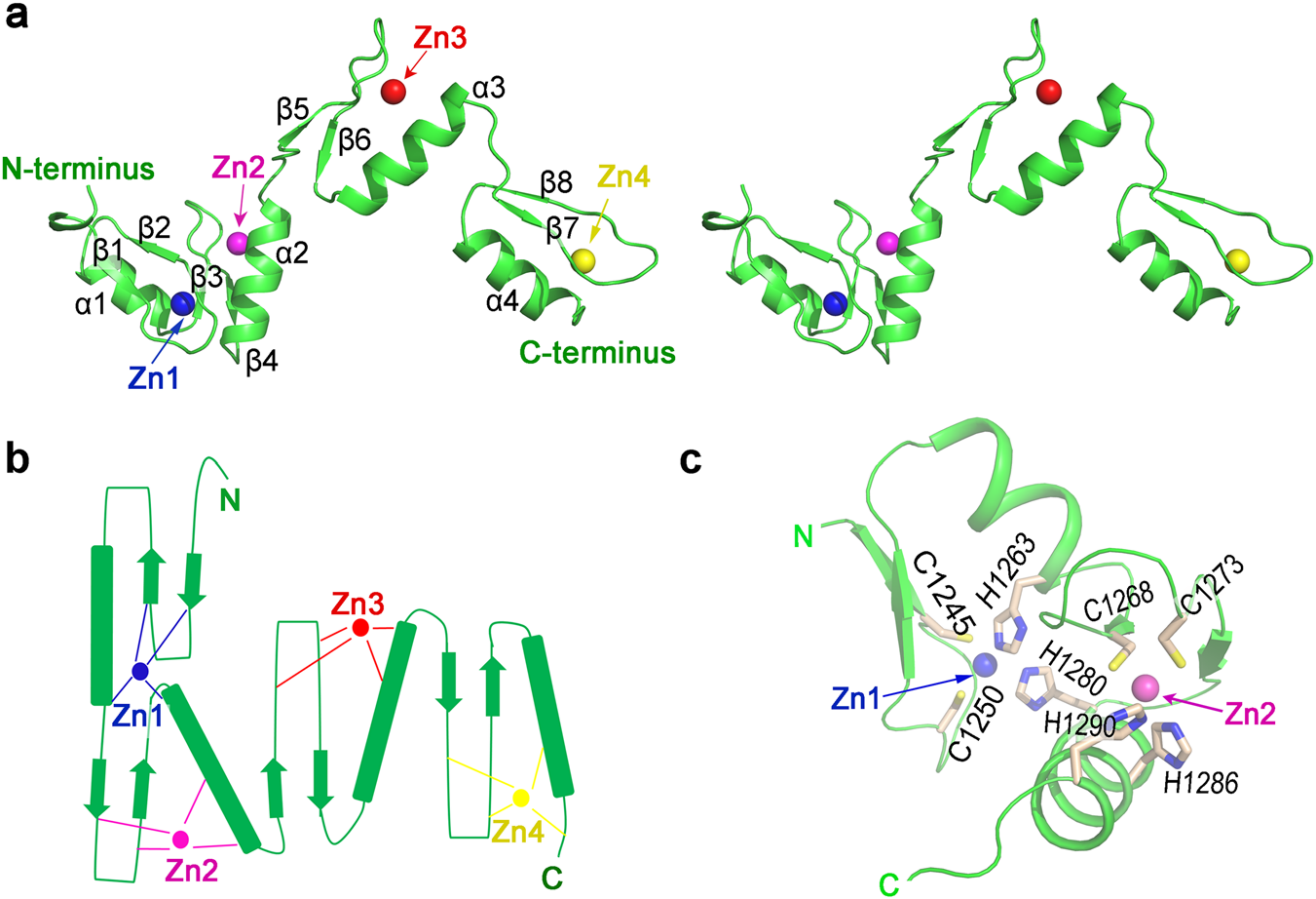
Crystal structure of the holo-REF6 ZnF domains. **a** Stereoview of the holo-REF6 structure. REF6 is in green color and the four Zn^2+^ ions (Zn1-4) are presented in blue, magenta, red, and yellow spheres, respectively. **b** The topology of REF6 contains a novel RCZ domain and two C2H2-type ZnF domains. **c** Close-up view of the RCZ structure. Residues C1245, C1250, H1263, and H1280 engage the first Zn^2+^ ion (Zn1), while residues C1268, C1273, H1286, and H1290 bind Zn2.

The holo-REF6^1223–1360^ structure contains four C2H2-type ZnF domains. ZnF3 and ZnF4 adopt a fold consisting of a canonical ββα domain^16–22^ in which a bound zinc ion is sandwiched between the α-helix and the two antiparallel β-sheets on the other side (Fig. 2a). Unexpectedly, the first two ZnF domains (ZnF1-2) are half-cross-braced. In particular, the Zn1 ion is tetrahedrally coordinated by two cysteines in strands β1 and β2, histidine H1263 in helix α1 from the ZnF1 domain, and histidine H1280 in helix α2 from the second ZnF2 domain (Fig. 2b). This particular Zn1 ion tightly connects ZnF1 and ZnF2 domains, leading to an overall compact structure (Fig. 2c). We submitted the coordinates of ZnF1-2 (residues 1239–1290) to the Dali server and found no similar structure in the Protein Data Bank (PDB), suggesting that the ZnF1-2 domain forms a novel zinc finger type. Most known cross-braced ZnFs, such as PHD, B-Box, RING, and HIT domains (Supplementary Fig. S3)^23^, use two coordinating residues from the first ZnF domain, and two residues from the second ZnF domain, which we denote as 2 + 2 type. The only other type of known half-cross-braced ZnFs is a cysteine-rich domain of rat brain PKC-γ (Supplementary Fig. S3f)^24^, which has one coordinating residue from the first ZnF and three from the second ZnF (1 + 3). To the best of our knowledge, the ZnF1-2 domain of REF6 represents a novel discovery of a half-cross-braced ZnF that has a 3 + 1 type coordination, which is distinct from other cross-braced ZnF domains. Therefore, we name this domain as a REF6-family-specific half-Cross-braced ZnF (RCZ) domain.

### The complex structure of REF6 with double-stranded DNA

**Fig. 3:**
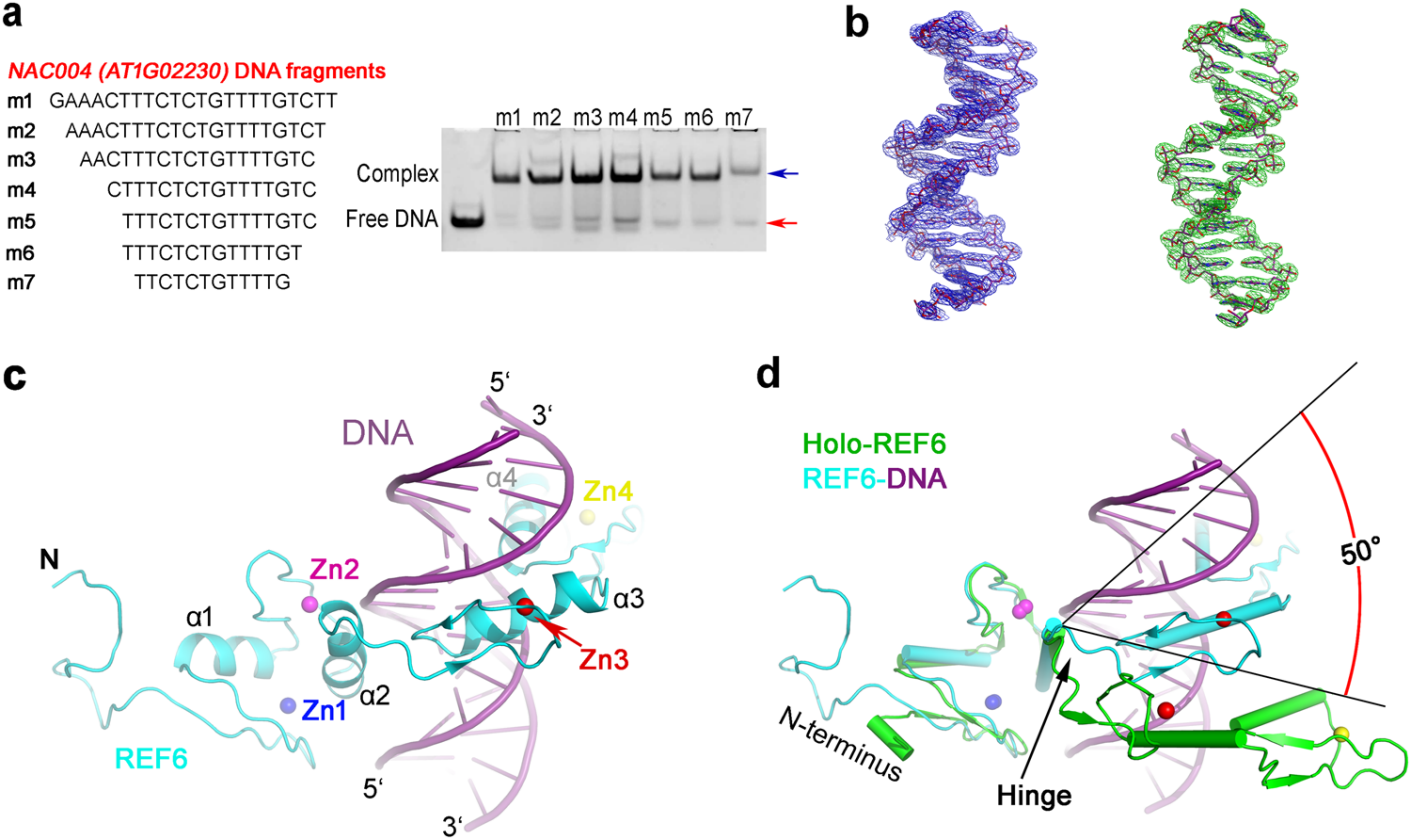
Structure of the REF6-DNA binary complex. **a** REF6 binds to *NAC004* dsDNA of different lengths with the same concentration. Positions of free dsDNA and protein-bound DNA are indicated by red and blue arrows, respectively. **b** The composite simulated-annealing sigma-A-weighted 2*mF*_o_ − *DF*_c_ (left) and sigma-A-weighted *mF*_o_ *−* *DF*_c_ (right) electron density maps are shown at 1.5*σ* and 2.5*σ*, respectively. **c** Ribbon representation of the REF6-DNA structure. REF6 and DNA are in blue and purple color, respectively. **d** Superposition of the holo-REF6 (green) and the REF6-DNA complex (REF6 in cyan and DNA in purple) structures. The coordinates of the RCZ domains from each were aligned. The last two ZnF domains showed a rotation of ~50° towards the dsDNA.

The ZnF domains of REF6 are essential for its recruitment to specific nucleosome sites via direct recognition of the CTCTGYTY motif^13,14^. To investigate the molecular mechanism of DNA recognition, we first screened a series of double-stranded oligonucleotides ranging from 12 to 22 bp of the *NAC004* (*AT1G02230*) gene, which contains a CTCTGTTT motif, and performed electrophoretic mobility shift assays (EMSA). The EMSA results indicated that the length of the DNA fragments had little effect on the binding (Fig. 3a).

Attempts to cocrystallize REF6 (residues 1223–1360) with different lengths of dsDNA resulted in a complex crystal with a 15-bp DNA fragment (m5). The structure was solved to a resolution of 2.7 Å using the SAD method by SHELX C/D/E (Supplementary Fig. S1d). In the asymmetric unit, one REF6, one dsDNA, two glycerol molecules, and 79 water molecules are modeled into the density map. Compared with holo-REF6, most of the acidic region is resolved in the complex structure. The electron density is well-resolved to allow unambiguous assignment of all the DNA nucleotides (Fig. 3b). Moreover, the composite simulated-annealing sigma-A-weighted *mF*_o_ − *DF*_c_ omit map clearly confirms the presence of a bound dsDNA (Fig. 3b). Consistent with this observation, the complex was eluted prior to the holo-REF6 which appeared as a single peak following gel filtration chromatography (Supplementary Fig. S4).

### dsDNA binding induces profound conformational changes

The binding of REF6 to DNA is important for its in vivo demethylase activity^13,14^. Our complex structure provides details of this interaction at the atomic level. Direct binding with the dsDNA strand involves insertion of the ZnF3 and ZnF4 helices into the major groove. Additionally, the β strands and the zinc ions lie on the outside, forming a tetrahedral structural unit that confers rigidity to the fingers (Fig. 3c). Surprisingly, only helices α3 and α4 insert into the DNA major groove (Fig. 3c). Helix α2 of the RCZ domain is situated above the major groove while helix α1 projects away from the DNA entirely. Furthermore, the four β-strands of the RCZ domain, with the exception of the C-termini of β4, also project away from the DNA.

The ZnF1 domain neighbors the acidic region and its surface is less electropositive than the other ZnFs (Supplementary Fig. S5). Moreover, the acidic region repels DNA. Taken together, this explains why the ZnF1 domain is not directly involved in DNA binding, whereas the ZnF2, ZnF3, and ZnF4 domains are. Most interactions between the ZnF2 domain and DNA occur on the phosphodiester backbone. Conformations of the holo-REF6 and REF6-DNA binary complex differ greatly with an overall RMSD of 5.3 Å (Fig. 3d and Supplementary Movie S1). Interestingly, the relative position of the ZnF3 and ZnF4 domains (ZnF3-4) does not change during DNA binding and can be viewed as a rigid body, despite the interface between them being only 270 Å^2^. Moreover, the RCZ and ZnF3-4 conformations in these two structures are similar (RMSD = 1.4 and 0.9 Å, respectively). A Dali server search of the ZnF3-4 domain structure identified the ZnF1-2 domain of the Wilms tumor protein (WT1, PDB 6B0R, *Z* = 8.3, RMSD = 2.0 Å)^25^ and the ZnF1-2 domain of Early growth response protein 1 (Egr1, PDB 1A1F, *Z* = 8.1, RMSD = 1.6 Å)^26^ as the closest structural homologs (Supplementary Fig. S6a). However, the sequence identity of these domains is less than 30%, indicating low homology. Moreover, only two ZnF domains of REF6 directly insert into the DNA major groove, compared with three or four ZnF domains in Egr1 and WT1, respectively. For the RCZ domain, four sheet-coil transitions (Fig. 1b) and DNA-induced conformational changes of the main chains are observed. Remarkably, structural comparison of holo-REF6 and the REF6-DNA binary complex reveals that the relative orientation of domains RCZ and ZnF3-4 undergoes a rotation of ~50° (Fig. 3d). Therefore, residues 1291–1293 act as a flexible hinge and dsDNA binding induces profound conformational changes of REF6. The shift of the integrated ZnF3-4 domain causes REF6 to form a ring-shaped clamp onto dsDNA (Supplementary Fig. S5a).

### Domains ZnF3 and ZnF4 are essential for dsDNA binding

**Fig. 4:**
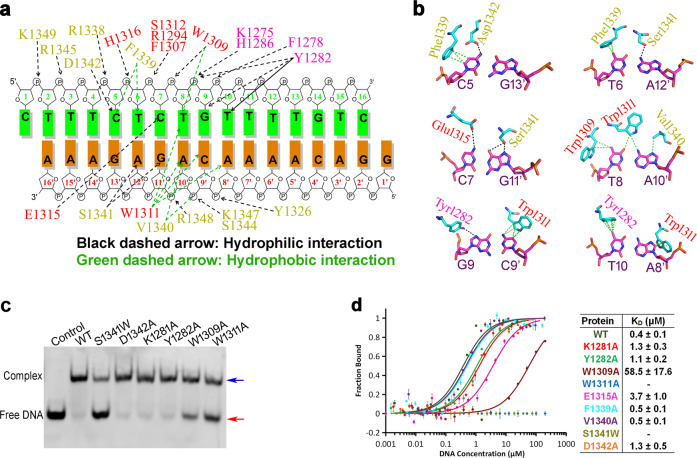
Interaction of REF6 and dsDNA. **a** Schematic representation summarizing the REF6−DNA interactions. The sequence of the dsDNA (*NAC004* fragment) used for crystallization is shown with two complementary strands. The residues involved in the interaction with the dsDNA are labeled in magenta, red, and yellow for domains ZnF2, 3, and 4, respectively. The green and black dashed arrows indicate hydrophobic and hydrophilic interactions, respectively. **b** Close-up view of the interaction between key residues and DNA bases. **c** EMSA of different REF6 mutants with the dsDNA. **d** Measurement of the binding affinity of the WT and mutant REF6 with dsDNA by MST. The experiments were repeated for three times.

The total buried interface between REF6 and dsDNA reaches 1077 Å^2^, indicating a strong interaction. There are many interactions at the interface between REF6 and dsDNA, including hydrogen bonds, electrostatic interactions, and hydrophobic interactions (Fig. 4a). Most hydrophilic interactions between REF6 and DNA occur on the phosphodiester backbone. The side chains of Lys1275, Tyr1282, His1286, Arg1294, Trp1309, Ser1312, His1316, Tyr1326, Arg1338, Ser1344, Arg1348, and Lys1349, and the backbone of Phe1278, form hydrogen bonds or electrostatic interactions with DNA phosphate groups (Supplementary Fig. S6b). Sequence-independent hydrophobic interactions with the DNA backbone occur via the side chains of Phe1277, Val1289, Phe1307, and Val1340 (Fig. 4a; Supplementary Fig. S6b and Supplementary Table S2). In addition, the side chains of Trp1309, Phe1339, and Val1340 interact with the bases through hydrophobic interactions. For sequence-dependent interactions, the side chain of Tyr1282 interacts with the base of G9 through a hydrogen bond and the base of T10 through hydrophobic interaction. The side chain of Trp1311 forms a hydrophobic network with the bases of T8, A8′, C9′, and A10′ (where ′ indicates the complementary strand). Meanwhile, the residues of Trp1309 and Trp1311 form a hydrophobic environment and together interact with the base of T8. The side chain of Glu1315 forms a hydrogen bond with the bases of C7. The side chain of Ser1341 forms two hydrogen bonds with the bases of G11′ and A12′. Moreover, the side chain of Asp1342 interacts with the base of C5 through a hydrogen bond (Fig. 4b).

To test the role of the above key residues, point mutants of REF6 were generated and circular dichroism (CD) experiments were conducted and confirmed that the mutants maintained the same secondary structural elements to that of WT (Supplementary Fig. S7). Meanwhile, EMSAs were performed to understand the qualitative characteristics of DNA binding among the REF6 mutants (Fig. 4c), while microscale thermophoresis (MST) experiments (Fig. 4d) were used to determine the equilibrium dissociation constants (*K*_D_). Consistently, mutants K1281A (*K*_D_ = 1.3 μM), Y1282A (*K*_D_ = 1.1 μM), and D1342A (*K*_D_ = 1.3 μM) exhibited reduced binding affinity to DNA compared with the WT (*K*_D_ = 0.4 μM), while mutation W1309A (*K*_D_ = 58.5 μM) exhibited a drastic decrease in binding affinity (Fig. 4d). Mutations W1311A and S1341W were generated in order to reduce or introduce large side chains, respectively. The result of which was the marked abrogation of DNA binding (Fig. 4c, d). Furthermore, we also measured the binding affinity to some weak interactions about the residues E1315A (*K*_D_ = 3.7 μM), F1339A (*K*_D_ = 0.5 μM) and V1340A (*K*_D_ = 0.5 μM). From the MST and EMSA data, mutations of the key residues in ZnF3 and ZnF4 domains dramatically decreased DNA binding. Together, these findings demonstrate that the ZnF3 and ZnF4 domains are essential for dsDNA binding.

### DNA methylation reduces the binding of dsDNA to REF6

**Fig. 5:**
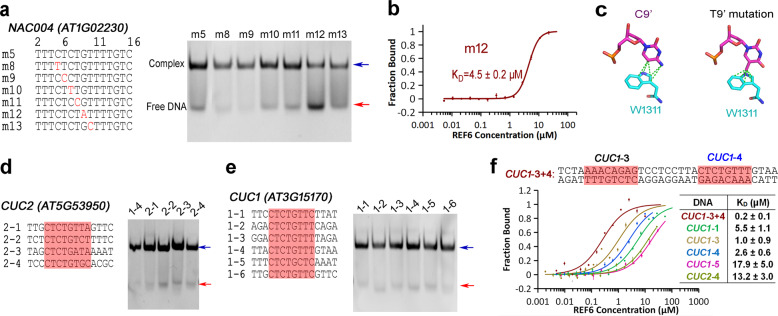
Binding of REF6 to different dsDNA fragments. **a** EMSA of REF6 with single-base mutations of the *NAC004* fragment. **b** MST measurement of the binding affinity of m12 (mutated G:C base pair with an A:T base pair) with REF6. **c** Modeling of the m12 and REF6 interaction. The mutation from C9′ (left) to T9′ (right) creates a steric clash with REF6. REF6 can bind to both *CUC2* (**d**) and *CUC1* (**e**) dsDNA fragments based on EMSA. **f** Comparison of the binding affinities of *CUC1* and *CUC2* with REF6. Note that *CUC1*-3 + 4 has two CTCTGYTY motifs.

For each strand of dsDNA that contains the CTCTGYTY motif, only five or six bases were directly involved in binding REF6 (Fig. 4a, b). To investigate the sequence specificity of REF6, we first mutated the core DNA sequence CTCTGT, which contributes crucially to REF6 binding (Fig. 5a). The binding affinity of REF6 to the G9A mutant (m12; *K*_D_ = 4.5 μM) (Fig. 5b) decreased over tenfold compared with the WT fragment (m5; *K*_D_ = 0.4 μM) (Fig. 4d). Structural analysis revealed that this was mainly due to steric hindrance caused by the C9′T mutation in the complementary strand, which introduced an additional methyl group that clashed with W1311 (Fig. 5c).

The fact that a single-base mutation affects binding suggests that DNA methylation might participate in recognition. Therefore, three different methylated modification sites of the *NAC004* DNA fragments were synthesized and EMSA experiments were performed. Consistent with the structural observation, the methylated dsDNA of m5-m9′C exhibited lower binding affinity than WT (Supplementary Fig. S8a), and the binding affinity of m5-m9′C (methylation at 9′C) to REF6 (*K*_D_ = 5.9 μM) decreased about 15-fold compared with the wild-type m5 fragment (*K*_D_ = 0.4 μM) (Supplementary Fig. S8b). Previous studies reported that no REF6 binding signal was detected in *CUC1*-1^13^. To uncover the reason, we analyzed the methylomes of *Arabidopsis thaliana*^27^. Methylation of this cytosine was found in the CHG methylome. We synthesized the *CUC1*-1 dsDNA with and without methylation. EMSA experiments (Supplementary Fig. S8c) confirm that DNA methylation reduces the binding of dsDNA to REF6 and that the dissociation constant of *CUC1*-1 m9′C is twice that of the unmodified *CUC1*-1 (Supplementary Fig. S8d). These findings confirm the hypothesis that DNA modification influences the recognition of DNA by REF6.

### The shape of DNA affects its binding to REF6

Our structural analyses indicate that only the bases of the CTCTGT motif are directly involved in binding REF6 (Fig. 4a, b). In *Arabidopsis*, *CUC1* and *CUC2*, which are functionally redundant genes, act as key regulators of boundary formation in cotyledons, sepals, and other organs^28,29^. Previous research revealed that REF6-ZnF domains bound strongly to *CUC1* locus, whereas *CUC2* had no REF6 binding signal^13^. Based on our REF6-DNA complex structure and the above mutagenesis experiments, we speculated that the CTCTGT sequence might be sufficient to bind to REF6. Therefore, we first searched the full-length gene of *CUC2* using the motif CTCTG and identified four sequences, three of which contain the CTCTGT motif (Fig. 5d). Our EMSA experiments showed that REF6 also could directly bind to all four *CUC2* and six *CUC1* motifs in vitro (Fig. 5d, e). To quantitatively compare the difference in binding affinity, we performed MST experiments and found that the affinities for most *CUC1* binding motifs were substantially higher than those for the *CUC2* motifs (Fig. 5f). The MST experiments showed that *CUC1*-5 (*K*_D_ = 17.9 μM) and *CUC2*-4 (*K*_D_ = 13.2 μM) exhibited weak binding affinities to REF6. On the other hand, the *CUC1*-3 (*K*_D_ = 1.0 μM) and *CUC1*-4 (*K*_D_ = 2.6 μM) motifs had strong REF6 binding affinities (Fig. 5f). Although all of the *CUC1* and binding motifs contained the CTCTGT motif, the affinities differed greatly. We, therefore, hypothesized that sequence-dependent conformations of DNA might confer the observed differences.

**Fig. 6:**
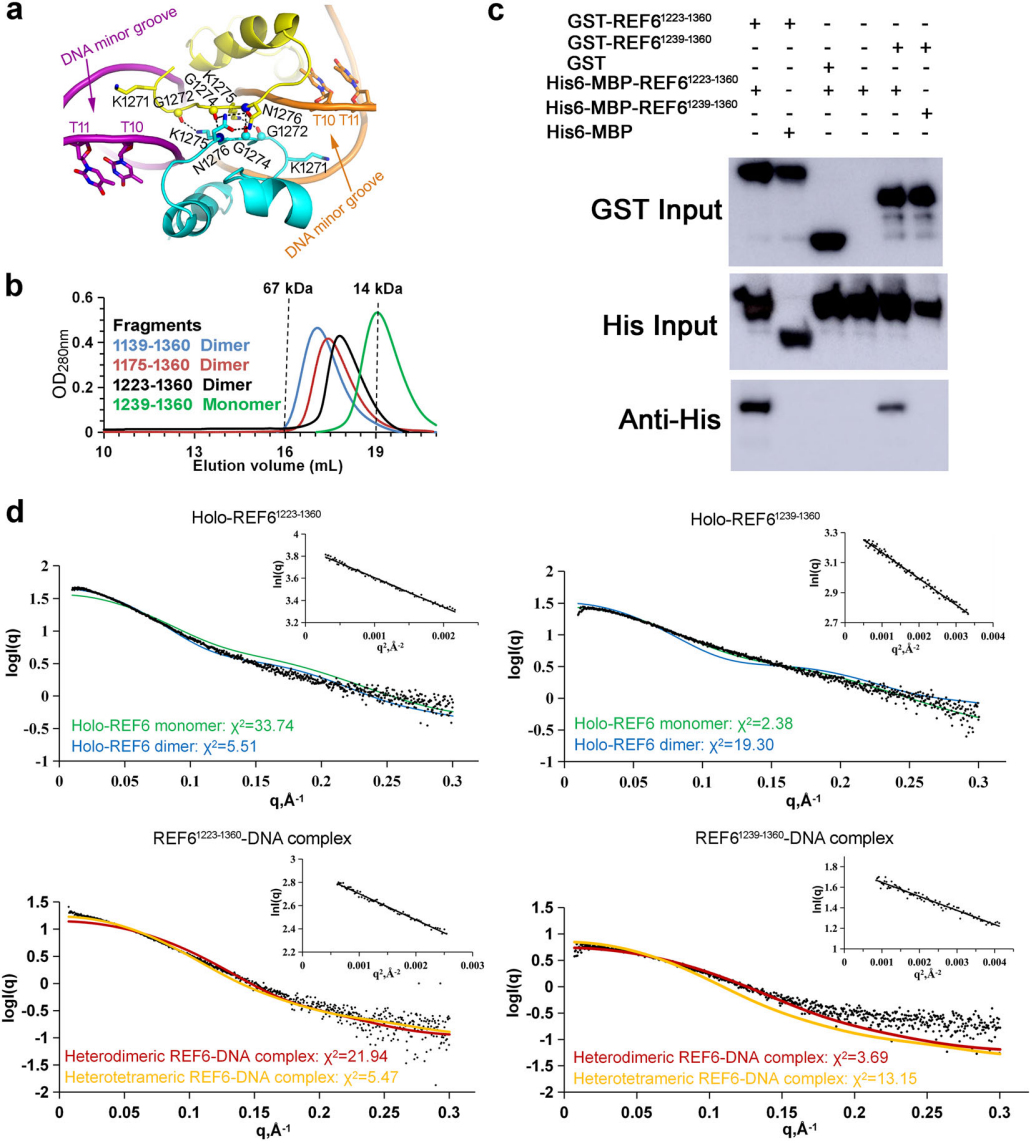
Self-association of REF6 with or without dsDNA. **a** The interface between two REF6 proteins in the complex structure. **b** Analytical gel filtration profiles of the REF6 fragments on a Superdex 200 10/300 GL column at 5.0 mg mL^−1^. The fragments of 1139–1360, 1175–1360, 1223–1360 and 1239–1360, all containing the four ZnF domains with different N-terminal extensions, are labeled in blue, red, black, and green, respectively. Elution volumes of the molecular mass standards are marked at the top of the panel. **c** Various fragments of purified GST-REF6 were used for GST pull-down of purified His-REF6 in the presence of NAC004 DNA fragment. Input and eluted proteins were analyzed by Western blot analysis with the anti-His antibody. **d** Comparison of SAXS experimental data and calculated scattering profiles. The inset shows the Guinier fits of 5 mg ml^−1^ holo-REF6 and REF6-DNA complex. Experimental data are represented in black dots. Holo-REF6 monomer (green), holo-REF6 dimer (blue), heterodimeric REF6-DNA complex (red), heterotetrameric REF6-DNA complex (yellow). Note that holo-REF6 dimer and heterotetrameric REF6-DNA complex models are built based on the structures obtained by the symmetric operation.

To test this hypothesis, we analyzed the bound DNA conformation in the REF6-DNA complex structure. Structural observation showed that the DNA was bent by 16° compared with the standard B-form DNA duplex (Supplementary Fig. S5b). The minor groove width (MGW) of dsDNA was calculated using the software CURVES+^30^. The results revealed that the MGW of each nucleotide in the complex structure varied greatly. In particular, the MGW in the middle of T10 and T11 decreased to the lowest value of 2.7 Å, less than half of the standard value of 5.7 Å (Supplementary Fig. S9a, b). The narrower minor groove widened the corresponding major groove and favored the insertion of the helices in REF6 to the major groove. We then calculated the theoretical MGW of dsDNA from *CUC1* and *CUC2* using the DNAshape server^31^. Consistent with the above experimental results, the six CTCTGYTY motifs in *CUC1* except *CUC1*-5 exhibited narrower MGW compared with the four CTCTG motifs from *CUC2* (Supplementary Fig. S9a). Moreover, further structural analysis revealed that Lys1271 from the neighboring REF6 inserted into the narrow minor groove (Fig. 6a). Consistently, the six motifs in *CUC1*, except *CUC1*-5, exhibited more negative electrostatic potential (EP) compared with the four motifs in *CUC2* (Supplementary Fig. S9b). The more negative EP enhanced the interaction with the positive Lys1271, thereby promoting the binding of REF6. Therefore, DNA shape, including MGW and EP, affects the binding of DNA to REF6.

### Self-association of REF6

To study the oligomeric state of holo-REF6 in our crystals, we utilized the Protein Interfaces, Surfaces and Assemblies (PISA) server^32^. The interaction between molecules from two neighboring symmetric units was not strong, with a buried interface area of 718 Å^2^. To determine the oligomeric state in solution, various REF6 truncations, including fragments 1139–1360, 1175–1360, 1223–1360, and 1239–1360, were generated. According to the elution volume from gel filtration chromatography, fragments of 1139–1360, 1175–1360, and 1223–1360 formed homodimers in solution, but fragment 1239–1360 existed as a monomer (Fig. 6b). Furthermore, GST pull-down experiments showed that GST-REF6^1223–1360^ directly interacted with His6-MBP-REF6^1223–1360^ in vitro, but not with GST or MBP alone (Fig. 6c). Therefore, longer fragments of REF6 interact with each other in solution. To further confirm the oligomeric states of REF6 in solution, small-angle X-ray scattering analysis (SAXS) was performed. As a complementary method to the high-resolution methods of X-ray crystallography, this provides a powerful tool for analyzing dynamic components in solution. We conducted SAXS using a concentration series from 0.5 to 5 mg mL^−1^, and the results revealed that the oligomerization of REF6 was concentration-dependent. At high concentration such as 5 mg mL^−1^, the dimeric form fits best for holo-REF6^1223–1360^, while the monomeric form fits best for holo-REF6^1239–1360^ (Fig. 6d).

**Fig. 7:**
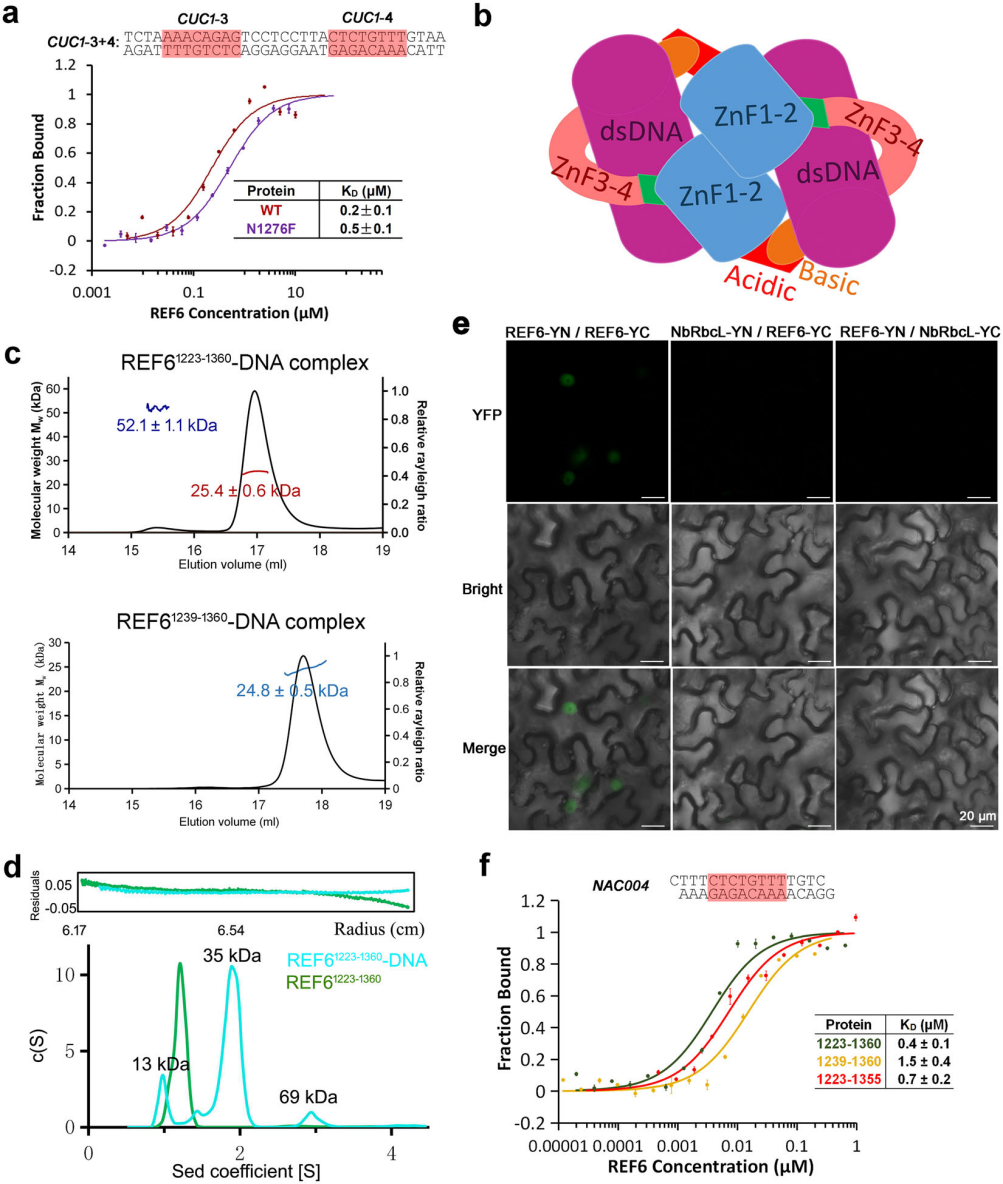
Cooperativity of REF6 enhances its binding to dsDNA. **a** The binding affinity of the mutation N1276F at the interface with *CUC1*-3 + 4 is lower than the WT. **b** Model of a heterotetrameric REF6-DNA complex. **c** The SEC-MALS results show the oligomeric states of REF6^1223–1360^-DNA complex and REF6^1239–1360^-DNA complex at 0.8 mg mL^−1^. **d** The oligomeric state of REF6^1223–1360^ by analytical ultracentrifugation assays at 1.0 mg mL^−1^. The c(s) distribution from SV analysis is shown. **e** BiFC assays showing self-association of the full-length REF6 in vivo. An unrelated chloroplast protein encoded by NbRbcL was used as a negative control^62^. **f** Loss of the acidic region or basic region obviously decreased the DNA-binding affinity of REF6 with *NAC004* dsDNA fragments.

In the complex structure, only one REF6-DNA heterodimer (one REF6^1223–1360^ and one m5 dsDNA fragment) was found in the asymmetric unit (Fig. 3c). Because the basic region in the C-termini and some of the residues in the N-terminal acidic region were not visible in the structure, we can not observe the interaction between the C-terminal basic region and the N-terminal acidic region within symmetric units directly. Meanwhile, the interaction between REF6 from two neighboring REF6-DNA complexes occurred between residues 1272–1276 in the ZnF2 domain with a small buried interface area of 234 Å^2^ (Fig. 6a). We analyzed the binding affinity of the mutant N1276F at the interface between two REF6 proteins. The affinity of the double-motif-containing *CUC1*-3 + 4 to WT was 2.5-fold higher than to the mutant N1276F (Fig. 7a). This result indicated that the residue Asn1276 participated in heterotetramer formation, i.e., the dimerization of two REF6-DNA complexes. The free energy of dissociation (Δ*G*^diss^) for the heterotetramer (two REF6-DNA complexes) was 20.3 kcal mol^−1^, calculated by the PISA server. The large positive value of Δ*G*^diss^ indicates that the dimerization of REF6^1223–1360^-DNA results in a heterotetramer, largely attributed to the dsDNA binding therein. Therefore, we can reasonably construct a model that describes an REF6-DNA heterotetramer by applying a two-fold symmetry operation (Fig. 7b). We further analyzed the oligomeric state of the REF6-DNA complex in solution. The SAXS results implied that the REF6^1223–1360^-DNA complex existed remarkably as a heterotetramer, but the heterodimer fits best to the profile for the REF6^1239–1360^-DNA complex at 5 mg ml^−1^ (Fig. 6d). Consistent with this observation, the molecular weight of the REF6^1223–1360^-DNA heterotetrameric complex was obviously larger than the holo-REF6^1223–1360^ dimer (Supplementary Fig. S4). The results from SEC-MALS showed that the REF6^1223–1360^-DNA complex formed two oligomeric states, the heterodimer and heterotetramer. Meanwhile, the REF6^1239–1360^-DNA complex only existed as a heterodimer (Fig. 7c). Furthermore, sedimentation velocity (SV) AUC analysis showed that the content of REF6-DNA complex heterotetramer in the REF6^1223–1360^-DNA complex was significantly higher than those in REF6^1239–1360^-DNA complex and REF6^1239–1355^-DNA complex (Fig. 7d and Supplementary Fig. S10). Importantly, chemical crosslinking supports this conclusion (Supplementary Fig. S11).

To further verify the oligomeric state in vivo, we performed bimolecular fluorescence complementation (BiFC) assay and confirmed that the full-length REF6 protein interacted with itself. An unrelated chloroplast protein encoded by NbRbcL was used as a negative control and did not interact with REF6 (Fig. 7e). Together, these results suggest that self-association exists for REF6 in vitro and in vivo.

### Cooperativity of REF6 enhances its binding to dsDNA

Neither the N-terminal acidic region nor the C-terminal basic region of REF6 ZnF domains was involved in binding dsDNA directly. However, the loss of either component substantially decreased its DNA-binding affinity. We compared the affinities of different REF6 fragments and found that the affinity of REF6^1223–1360^ was substantially higher than those of REF6^1239–1360^ and REF6^1223–1355^ (Fig. 7f). It is also consistent that the loss of the N-terminal acidic region in REF6^1239–1360^ greatly decreased its interaction with REF6^1223–1360^ (Fig. 6c). Meanwhile, no interaction was observed between GST-REF6^1239–1360^ and His6-MBP-REF6^1239–1360^ in vitro. Thus, the N-terminal acidic region is essential for heterotetramer formation. In addition, the mutated residue N1276F at the interface between two REF6 proteins decreased the binding affinity compared with the WT (Fig. 7a). The affinity differences might be due to the missing or mutated residues participating in the formation of the heterotetramer. Thus, the results imply the existence of cooperativity in binding dsDNA and that the self-association of REF6 enhances binding to dsDNA.

Furthermore, we compared the affinities of *CUC1*-3, *CUC1*-4, and *CUC1*-3 + 4 which included different numbers of REF6 target motifs. In *CUC1*-3 + 4, the two CTCTGYTY motifs are separated by nine base pairs and are found in the two complementary strands of *CUC1* (Fig. 5f). The affinity of the double-motif-containing *CUC1*-3 + 4 to REF6 was 5- and 13-fold higher than those of *CUC1*-3 or *CUC1*-4 separately (Fig. 5f), revealing that recognition of the CTCTGYTY motif by REF6 may be cooperatively modulated by the number of neighboring motifs. The cooperative binding of several proteins to one DNA has been reported for *YUC3* by REF6 ^14^ and other proteins^33^. Moreover, the large Hill coefficient of 1.9 for the *CUC1*-3 + 4 calculated using the Hill model (Supplementary Fig. S12a) further confirmed the cooperativity, while both Hill coefficients were less than 1 for separate experiments using *CUC1*-3 or *CUC1*-4. Meanwhile, the cooperativity of REF6^1223–1360^ was higher than that of the mutant N1276F (Supplementary Fig. S12b). These cooperative differences further confirmed the missing or mutated residues participating in the formation of the heterotetramer. Overall, the binding cooperativity may explain the differential binding ability of REF6 to different numbers of CTCTGYTY motifs.

## Discussion

The ZnF domains found in histone demethylases are primarily involved in binding histones or ubiquitination. Very few can bind DNA directly (ePHD2 in the PHF6 protein^34^, the CXXC domain of JHDM1 subfamily^35^, and the ZnF domains in the REF6 protein subfamily). To the best of our knowledge, reports on complex structures of C2H2 Zinc fingers with DNA are rare for histone demethylases. Here we present a new mechanism of histone demethylase recruitment to specific chromatin sequences. These results provide structural insights into the recognition mechanism of REF6 and its cognate DNA motif, including that the bases and DNA backbones interact with different ZnF domains to further enhance binding specificity. The flexible hinge region allows for reorientation of the ZnF3-4 domain in order to facilitate insertion into DNA (Fig. 3d), resulting in a more compact conformation compared with that of holo-REF6 (Supplementary Fig. S5a).

We show that the RCZ domain adopts a novel fold of the half-cross-braced ZnF protein family. All other known cross-braced ZnFs have two or three coordinating residues cross-braced from the second ZnF domain^23,24^. In the RCZ domain, only one residue is cross-braced. This finding may be connected with the specific recognition mechanism of the REF6 family. Sequence alignment from different plant species shows that the residues in the C-termini of the ZnF2 domain are conserved (Fig. 1b). The conservation suggests their functional importance and is consistent with our structural findings. For the RCZ domain in the DNA-bound structure, only the C-termini of β4 and α2 bind the phosphodiester backbone. Thus, the RCZ domain serves to stabilize DNA binding, but does not function in base recognition. Moreover, compared with the ZnF3-4 domain, the RCZ domain undergoes a remarkable conformational change upon binding (Fig. 3d) that involves four sheet-coil transitions (Fig. 1b) and a rotation of the N-terminus. Furthermore, the interaction residues between two neighboring REF6 proteins in the solved complex structure, as well as Lys1271 which can insert into the narrow minor groove, are all from the RCZ domain. This reveals an important regulatory function in binding DNA. Phylogenetic analysis shows that the RCZ domain might exist in plants only, suggesting a lineage-specific role.

Based on our structures, only the three-base CTC motif is directly recognized by the ZnF4 domain of REF6. Like ZnF4, the ZnF3 domain is positioned in the DNA major groove. It improves the binding affinity via many hydrophilic interactions with the phosphodiester backbone and hydrophobic interactions between Trp1309 and Trp1311 with nucleic acid bases. A previous study demonstrated that REF6 bound to *CUC1* in vivo, with the exception of *CUC1*-1, but not *CUC2*^13^. However, our in vitro experiments confirmed that four CTCTG motifs from *CUC2* bound REF6 directly. Moreover, the accurate binding experiments further showed that the affinity of all six *CUC1* and four *CUC2* motifs differ greatly (Fig. 5f), revealing the diversity of binding motifs. This raises the question of how REF6 achieves its substrate specificity in vivo due to the presence of 10 million such motifs in the *Arabidopsis* genome. When the oligonucleotide length is shorter than 12 bp, the binding affinity of REF6 decreases dramatically. For example, the binding affinity of the 10-bp sequence TTCTCTGTTT (*K*_D_ = 483 μM), which contains all of the interactions found in the structure, is very low compared with the 16-bp motif (*K*_D_ = 0.4 μM), revealing that DNA conformations are involved in DNA recognition.

Several studies have suggested that DNA shape, including MGW and electrostatic potential, contributes to specific DNA recognition^33,36,37^. Our experiments show that the CTCTGTTTT motif has the highest binding affinity, while other CTCTGYTY motifs are weaker. Thus, our results can explain the finding that only 15% of CTCTGYTY motifs are within REF6 target sites^13^. The theoretical MGW and electrostatic potential reveal that *CUC1*-3 and *NAC004* DNA, both with a TTTT sequence 3′ to CTCTG, have the narrowest minor groove and the most negative electrostatic potential (Supplementary Fig. S9) in the middle of T10 and T11. Meanwhile, the dissociation constant of REF6 towards the T-tract mutant (m13; *K*_D_ = 10.6 μM; Supplementary Fig. S9d) decreased over 25-fold compared with the WT m5 fragment (*K*_D_ = 0.4 μM). These results are consistent with the report that T-tract sequences tend to form a narrow minor groove^38,39^. Therefore, the narrower minor groove is an intrinsic structural feature of the DNA sequence. In addition, the feature may become further enhanced by the binding of REF6 as the value in the complex structure is smaller than the theoretical value (Supplementary Fig. S9a). Except for the role in widening the corresponding major groove and favoring the insertion of the REF6 helices, the specific DNA shape will favor the binding of Lys1271 using its more negative electrostatic potential. Thus, we suggest that shape readout of DNA structure may be a hallmark of the REF6 protein subfamily and constitutes a mechanism for REF6’s selectivity towards CTCTGYTY motifs.

Interestingly, we found that REF6 displays positive cooperativity. In addition, the ZnF3 domain might mediate self-association similar to the closest structural homolog, the ZnF1 in the Wilms tumor protein^25^ (Supplementary Fig. S6a). In both the holo- and DNA-bound REF6 structures, only one REF6 molecule was solved. However, the free energy of assembly, in vitro, and in vivo experimental results showed the existence of dimerization between REF6 fragments and heterotetramer formation with bound DNA. The acidic region and basic region are found at both termini of REF6 ZnF domains (Fig. 1a). The basic region is especially conserved in many plant species (Fig. 1b), revealing its functional importance. The acidic region appears to be nonconserved, but many continuous aspartic acid or glutamic acid residues are also found in the upstream region of the ZnF domains in many plant species. Although it is not resolved in the structures, the basic region was speculated to interact with the acidic region of a neighboring REF6 molecule (Fig. 7b). Missing or mutated residues participating in the formation of the heterotetramer substantially decreased its DNA-binding affinity (Figs. 6c, 7a). Moreover, the affinity of the double-motif-containing *CUC1*-3 + 4 was dramatically higher than those of the *CUC1*-3 or *CUC1*-4 fragments separately (Fig. 5f). Additionally, the shape of the binding curve is steeper for the longer construct compared with the short one (Supplementary Fig. S12). These results suggest the existence of cooperativity in binding dsDNA and that the self-association of REF6 greatly enhances the affinity. Thus, the REF6-DNA complex is able to form a heterotetramer, but the ratio is low. As one REF6 molecule interacts with dsDNA by forming a semi-circular structure across ZnF2-4 (Fig. 3c), two REF6-dsDNA complexes may dimerize to form a heterotetramer that encircles the oligonucleotides completely (Fig. 7b). The result of which substantially enhanced the binding stability. This cooperativity can explain the fact that REF6 tends to bind CTCTGYTY-motif clusters^13^.

DNA methylation is one of the most well-studied epigenetic marks. From our structures and in vitro experiments, we find that DNA methylation at specific sites disfavors the binding of REF6 to DNA. Our discovery of *CUC1*-1 methylation in the methylomes of *Arabidopsis* explains a previous report that no REF6 binding is detected when using a *CUC1*-1 substrate^13^. Recently, it is also reported that other factors, such as chromosome states, binding partners, the number of binding motifs, and even dependence on transcription factors, may influence the recognition by REF6 in vivo^40–44^. Take the case of CTCTGYTY-motif clusters, due to the high affinity from cooperativity, REF6 can tolerate sequence variation in the CTCTGYTY motif and thus might bind many kinds of motifs. All of the features found in our study, such as DNA methylation, MGW, and cooperativity, can contribute to the recognition of diverse sequence motifs. Thus, our results explain the finding that about 80% of REF6-targeted DNAs contain the CTCTGYTY motif and only 15% of CTCTGYTY motifs in the *Arabidopsis* genome overlapped with REF6-binding sites^13^.

In summary, these results uncover new recognition and recruitment mechanisms for histone demethylation. Despite the similarity of the REF6 residues at the interface compared with the complex structure of REF6 ZnF2-4 with dsDNAs^45^ (Supplementary Fig. S13), our structures and data reveal that REF6 recognizes dsDNA through diverse mechanisms, such as base and shape readouts, cooperativity of protein and binding motifs, and DNA methylation. Our findings not only provide logical explanations for the selective recognition of selected CTCTGYTY motifs by REF6, but also reveal the crosstalk between DNA modification and histone modification. Furthermore, the high affinity of the REF6 tandem ZnF domains in binding DNA, and/or in combinations with other zinc finger motifs, might be used as a tool for specific gene regulation interventions under different cell contexts or stimuli, such as curing or preventing diseases including cancers, diabetes, cardiovascular diseases, and viral infections.

### Notes on the complex structures of REF6 ZnF2-4 with DNAs

When we were preparing our manuscript, another group released complex structures of REF6 ZnF2-4 with methylated dsDNAs in the PDB (6JNN, 6JNM, and 6JNL)^45^. A quick comparison with our structures indicates that the binding interface between REF6 and DNA bases are similar. However, the sequence lengths of protein and nucleic acid in our complex structures are significantly longer. Thus, the narrow minor groove, the shape readout mechanism, and the cooperativity of REF6 were not observed in those structures.

Furthermore, our structures reveal that the ZnF1 is critical in stabilizing the conformation of the ZnF1−2 and important for the self-association and synergy of DNA recognition. Moreover, no holo-REF6 structures were reported. Our holo-REF6 structure also revealed a large conformational change required for the ZnF3-4 to bind DNAs. Thus, the major conclusions in our manuscript are not obtained in those structures. We believe our structures are more physiologically relevant than the aforementioned ZnF2-4 structures.

## Materials and methods

### Cloning, protein expression, and purification of REF6

Constructs of *Arabidopsis thaliana* REF6 were subcloned into either a modified pET28a vector encoding a His6-MBP fusion tag or the pGEX-4T-2 expression vector^33,46^. Both the MBP and the GST fusion tags are removed from the REF6 constructs by a tobacco etch virus (TEV) protease cleavage site. All mutants of REF6 were created using site-directed mutagenesis and verified by DNA sequencing. The plasmids were transformed into *Escherichia coli* BL21 (DE3) cells. Cells were grown in Luria-Bertani (LB) medium at 37 °C until OD_600_ reached 0.8–1.0 and the culture was then supplemented with 100 μM ZnCl_2_, and protein overexpression induced at 18 °C by 0.2 mM isopropyl *β*-d-1-thiogalactopyranoside. After 12 h, cells were harvested by centrifugation at 4000 × *g* for 10 min at 4 °C, and then resuspended in a lysis buffer (20 mM Tris pH 7.3, 1 M NaCl, 2 mM β-mercaptoethanol), replenished with 0.1% (v/v) Triton X-100 and 1 mM Phenylmethanesulfonyl fluoride (PMSF) (Invitrogen). Cells were lysed by sonication and clarified by centrifugation at 18,300 × *g* for 45 min. The lysate was filtered through a 0.45 μm filter membrane to remove cell debris before being loaded onto a Ni^2+^-chelating column or GST affinity column (GE Healthcare). The fusion tag was cleaved using TEV protease at 4 °C for 6–8 h when necessary. The proteins were further purified by size exclusion chromatography. The peak fractions were collected and pooled, and the purity was assessed by SDS-PAGE. The proteins were concentrated to 10–15 mg mL^−1^ before setting up for crystallization. The REF6-DNA complex was obtained by directly incubating the REF6 protein with an excess (1:1.1) of the specific DNA substrate in 20 mM MES (pH 6.0), 150 mM NaCl, 2 mM β-mercaptoethanol for 2 h on ice. The complex was further concentrated to 6 mg mL^−1^ for crystallization.

### Crystallization and data collection

Crystallization trials of the holo-REF6 and the REF6-DNA complex were performed with multiple commercial crystallization kits in 48-well plates using the hanging drop vapor diffusion method. The DNA oligonucleotides were prepared by annealing two complementary oligonucleotides (5′-CTTTCTCTGTTTTGTC-3′ and GGACAAAACAGAGAAA). Briefly, a mixture of the two oligos was incubated at 95 °C for 5 min and then allowed to cool down slowly to 20 °C in 2 h. The 15-bp duplex DNA with single-nucleotide overhangs at the 5′-ends was added in three-fold molar excess to 368 μM (6 mg mL^–1^) of REF6. High-quality holo-REF6 crystals were grown in a reservoir solution containing 12% PEG 20000, 100 mM MES, pH 6.5, at 18 °C, and the complex crystals were obtained in 100 mM HEPES (N-(2-hydroxyethyl) piperazine-N’-(2-ethanesulfonic acid)), pH 7.5, 26% PEG 400, and 100 mM CaCl_2_ at 18 °C. All crystals were cryoprotected in the mother liquor supplemented with 25% (v/v) glycerol and flash-frozen in liquid nitrogen before data collection. The data were collected on beamlines BL17U1 and BL19U1 at the Shanghai Synchrotron Radiation Facility (SSRF). Data collection statistics are summarized in Supplementary Table S1.

### Structure determination

Structures of holo-REF6 and REF6-DNA complex were both solved by using the SAD method. The anomalous signals in the data were strong as analyzed by the SHELX C program^15^, indicating the existence of zinc atoms. For holo-REF6 in space group *P*4_1_, four initial Zn sites were found by the program SHELX D with a CC_weak_/CC_all_ of 26.5/43. A crude partial model with four α-helices and eight β-strands in 102 residues was generated by SHELX E^15^ and the figure of merit reached 0.706 with a CC_part_ of 48.55%. The model was further extended using AUTOBUILD^47^ followed by manual model building using COOT^48^ and refinement using REFMAC5^49^ iteratively. The native dataset of the crystal grown at 18 °C was then used and the structure was refined finally to 1.57 Å resolution with an *R*_work_ of 21.1% and an *R*_free_ of 23.4%. For the REF6-DNA complex, we failed to find the phase with molecular replacement using the holo-REF6 coordinate. The complex structure was then solved by the SAD method using the anomalous signals of zinc atoms. The REF6-DNA complex crystal was grown in space group *P*3_2_21. Four initial Zn sites were found by SHELX D. A partial model with three α-helices and four β-sheets in 89 residues was generated by SHELX E^15^ and the figure of merit reached 0.695 with a CC_part_ of 22.62%. The model was further built in program Buccaneer^50^ to an *R*_work_/*R*_free_ of 0.41/0.47. The primitive density map showed a clear outline of nucleic acids. At this point, the standard structure of a B-form nucleic acid was fitted into the electron density map. After iterative manual model building in COOT and refinement with REFMAC5, the structure was refined to 2.7 Å resolution with an *R*_work_ of 22.1% and *R*_free_ of 25.7%.

All structural figures in this article were prepared using PyMOL (version 1.8.0.0, Schrödinger LLC)^51^.

### Gel filtration chromatography

Purified recombinant REF6 proteins were applied to gel filtration columns (Superdex-200, GE Healthcare) equilibrated with a buffer containing 20 mM HEPES, pH 7.3, 500 mM NaCl, and 2 mM β-mercaptoethanol. Peak fractions were collected and visualized by SDS-PAGE followed by Coomassie Bright Blue staining.

### Electrophoretic mobility shift assays

Double-stranded DNAs used for EMSA were prepared by annealing two complementary oligonucleotides. Briefly, a mixture of the two oligos was incubated at 95 °C for 5 min and then allowed to cool down slowly to 20 °C in 2 h. About 30 μM protein was incubated with 10 μM dsDNA in a 20 μL reaction mixture (containing 20 mM Tris pH 8.0, 100 mM NaCl, 2.5 mM MgCl_2_, 0.1% CA-630, 10% glycerol, 1 μM ZnSO_4_, 1 mM Dithiothreitol(DTT))^13^ for 1 h at 4 °C and 4 μL of the mixture was then separated on a 10% native polyacrylamide gel in 0.5× TBE buffer (45 mM Tris, pH 8.0, 45 mM boric acid, 1 mM ethylenediaminetetraacetic acid (EDTA), pH 8.0) at 180 V for about 30 min. The DNAs were visualized by staining with ethidium bromide.

### Microscale thermophoresis

The binding affinities between dsDNA oligonucleotides and REF6 were measured using microscale thermophoresis (MST). The oligonucleotides were labeled with 6-carboxyfluorescein (FAM) and annealed slowly from 95 to 20 °C similar to the oligos used for cocrystallization. Wild-type and mutated REF6 proteins were mixed with dsDNA in a buffer containing 20 mM HEPES, pH 7.3, 150 mM NaCl, 0.2 mM TCEP and 0.5% CA-630 (Sigma: I8896). After incubation for 30 min, the samples were loaded into silica capillaries and temperature-induced fluorescence changes were measured on a Monolith™ (NanoTemper) at 25 °C by using 20% LED-power and 40% MST-power. Data analyses were performed by using the NTAnalysis software (NanoTemper Technologies). The experiments were repeated three times using different protein preparations.

### DNA shape analysis and electrostatic potential calculation

Bound DNA conformation in the crystal structure was analyzed with CURVES+ ver.1.31^30^. All theoretical width and electrostatic potential of the minor groove for DNA motifs in the *CUC1*, *CUC2*, and *NAC004* were calculated using the DNAshape server^31^.

### Circular dichroism analysis

The circular dichroism (CD) signals of the samples were measured on the 4B8 beamline at the Beijing synchrotron radiation facility. Spectra were collected at 1 nm intervals over the wavelength ranging from 260 to 175 nm in a 0.005 cm optical path length at 25 °C. Protein samples were prepared at a concentration of about 10 mg mL^−1^. A pure solvent baseline collected with the same cell was subtracted. All spectra data were processed using the CD tool software package^52,53^. The differential absorbance of polarized lights (mdeg) was converted into the per residue molar absorption unit, delta epsilon (Δ*ε*) in M cm^−1^, by normalization with respect to polypeptide concentration and path length.

### Small-angle X-ray scattering

Small-angle X-ray scattering measurements were performed on beamline BL19U2 at the SSRF following previously published methods^54,55^. Briefly, all proteins were subjected to size exclusion chromatography in a buffer containing 20 mM HEPES, pH 7.3, 500 mM NaCl, 2 mM β-mercaptoethanol. The REF6-DNA complex was dialyzed in a buffer containing 20 mM MES (pH 6.0), 150 mM NaCl, and 2 mM β-mercaptoethanol. Various concentrations of protein were used, and the data were collected at 1.03 Å with a distance of 2.68 m from the detector. Individual data were processed by RAW^56^. The scattering data from the buffer alone were measured before and after each sample measurement, and the average of the scattering data was used for background subtraction. Data collection statistics are summarized in Supplementary Table S3^57^.

### Bimolecular fluorescence complementation assay

BiFC measurements were performed as described^58^ using a Zeiss LSM710 confocal microscope at 514 nm. The full-length REF6 was cloned into the pSPYCE and pSPYNE BiFC vectors. The constructs were transfected into *A. tumefaciens* strain GV3101. The transfected bacteria were used to infiltrate the lower epidermis of tobacco leaves. After 72 h, the fluorescence signals were measured.

### GST pull-down assay

REF6 constructs fused with His6-MBP or GST-tags were first purified using the appropriate affinity columns. Two hundred micrograms of His6-MBP-REF6 was incubated with GST-REF6 for 6 h at 4 °C in a 500 μL buffer of 20 mM MES (pH 6.0), 150 mM NaCl, 2 mM β-ME and 0.1% (v/v) Triton X-100 in the presence of glutathione agarose beads (GE). The resin was extensively rinsed with the same buffer to remove unbound or nonspecifically bound proteins. Proteins left on the beads were separated by SDS-PAGE and analyzed by Western blotting.

### Analytical ultracentrifugation

Sedimentation velocity (SV) analytical ultracentrifugation experiments were performed in a Beckman/Coulter XL-I analytical ultracentrifuge using double-sector or six-channel centerpieces and sapphire windows. The proteins were further purified by size exclusion chromatography in buffer containing 20 mM HEPES (pH 7.3) and 200 mM NaCl. The REF6-DNA complex was obtained by directly incubating the REF6 protein with an excess (1:3) of the specific DNA substrate in 20 mM MES (pH 6.0), 150 mM NaCl, for 2 h on ice. The proteins were concentrated to 1 mg mL^−1^ before the experiments. SV experiments were conducted at 42,000 rpm and 4 °C using interference detection and double-sector cells loaded. The data were analyzed using the SEDPHAT and SEDFIT programs^59,60^.

### Chemical crosslinking

The fragments of REF6^1223–1360^ and REF6^1239–1360^ were purified using affinity columns. Fifteen micrograms of REF6^1223–1360^ and REF6^1239–1360^ was incubated with glutaraldehyde at different concentrations in a 100 μL reaction volume. After incubation for 10 min at 25 °C, the crosslinking reaction was quenched with 1 M Tris, to produce a final concentration of 50 mM, and then incubated for 10 min at 25 °C. The samples were analyzed by SDS-PAGE followed by Coomassie Bright Blue staining^61^.

## Supplementary information

supplemental Information

Validation report

Validation report

supplemental Movie S1

## Data Availability

Atomic coordinates and structure factors for the reported crystal structures have been deposited with the Protein Data bank under accession numbers 6A57 and 6A58.

## Supplementary information

**Supplementary Information** accompanies the paper at (10.1038/s41421-020-0150-6).
