# Supplementary material for: Crystal structures of REF6 and its complex with DNA reveal diverse recognition mechanisms
Source: Cell Discov. 2020 Mar 31;6:17. doi: 10.1038/s41421-020-0150-6 (PMC7105484; doi:10.1038/s41421-020-0150-6)
Supplement: Supplementary file 1 — supplemental Information [file 41421_2020_150_MOESM1_ESM.docx]

**Supplemental Information**

**Crystal structures of REF6 and its complex with DNA reveal diverse recognition mechanisms**

Zizi Tian^1^, Xiaorong Li^1^, Min Li^1^, Wei Wu^1^, Manfeng Zhang^1^, Chenjun Tang^1^, Zhihui Li^1^, Yunlong Liu^1^, Zhenhang Chen^1^, Meiting Yang^1^, Lulu Ma^1^, Cody Caba^2^, Yufeng Tong^2^, Hon-Ming Lam^3^, Shaodong Dai^4^, Zhongzhou Chen^1,^*

^1^ State Key Laboratory of Agrobiotechnology and Beijing Advanced Innovation Center for Food Nutrition and Human Health, College of Biological Sciences, China Agricultural University, Beijing 100193, China

^2^ Department of Chemistry and Biochemistry, University of Windsor, Windsor, Ontario N9B 3P4, Canada

^3^ School of Life Sciences and Center for Soybean Research of the State Key Laboratory of Agrobiotechnology, The Chinese University of Hong Kong, Shatin, N.T., Hong Kong SAR

^4^ Department of Pharmaceutical Sciences, Skaggs School of Pharmacy and Pharmaceutical Sciences, University of Colorado Anschutz Medical Campus, Aurora, CO 80045

Running title: Crystal structures of the ZnF domains of REF6

* To whom correspondence should be addressed. Tel: 86-10-62734078; Fax: 86-10-62734078; Email: [chenzhongzhou@cau.edu.cn](mailto:chenzhongzhou@cau.edu.cn)


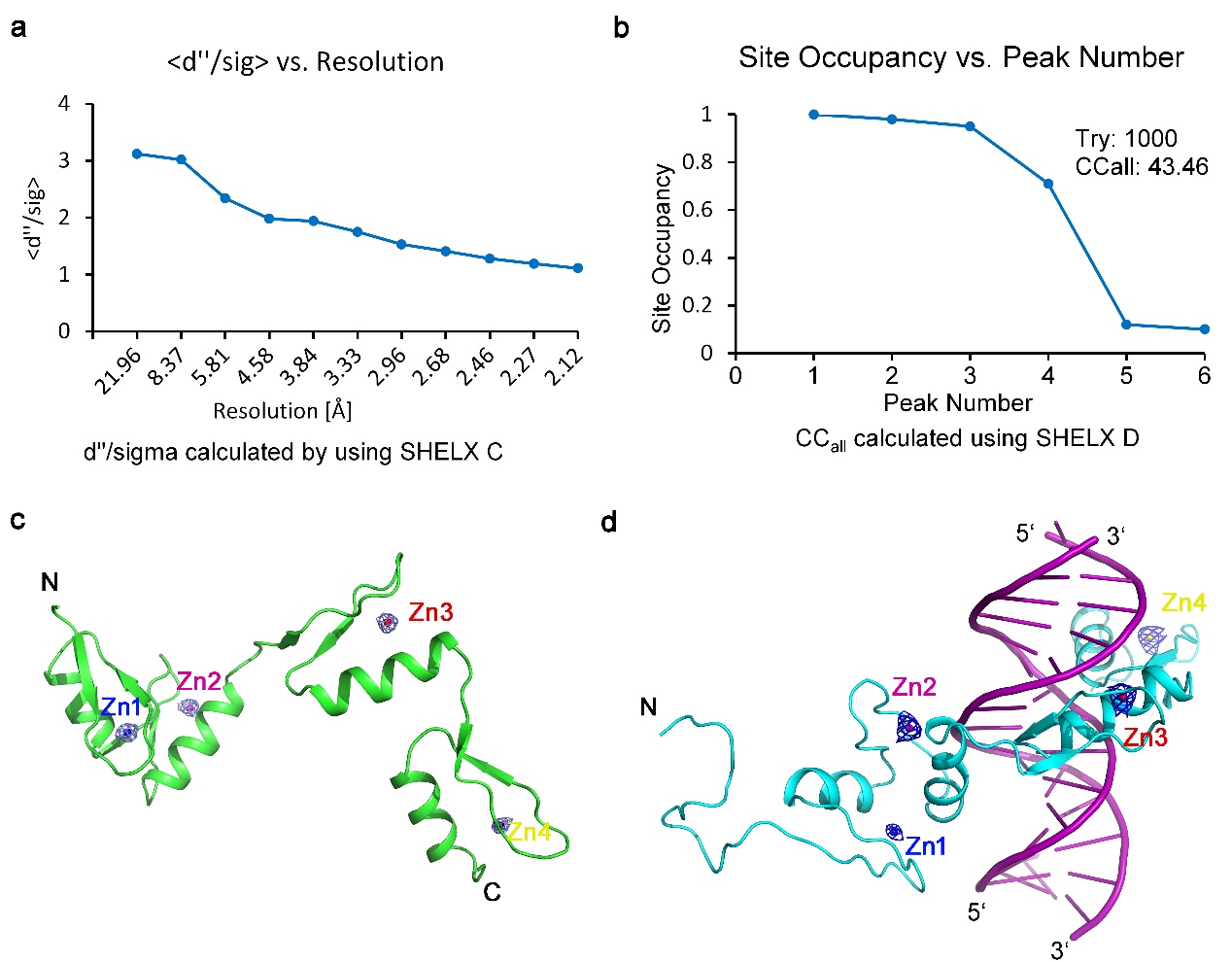


**Figure S1**. **The existence of four anomalous atoms found by the SHELX program**[**^1^**](#_ENREF_1)

The results of programs SHELX C (a) and SHELX D (b). The figure of merit (FOM) was 0.706 for the solution of SHELX E. For the holo-REF6 (c) and REF6-DNA complex (d), the blue meshes were contoured at 5.5 σ and 4.5 σ, respectively. The superimposed anomalous difference Fourier maps greatly helped identify the locations of four zinc atoms, and facilitated sequence assignment in the model building process.


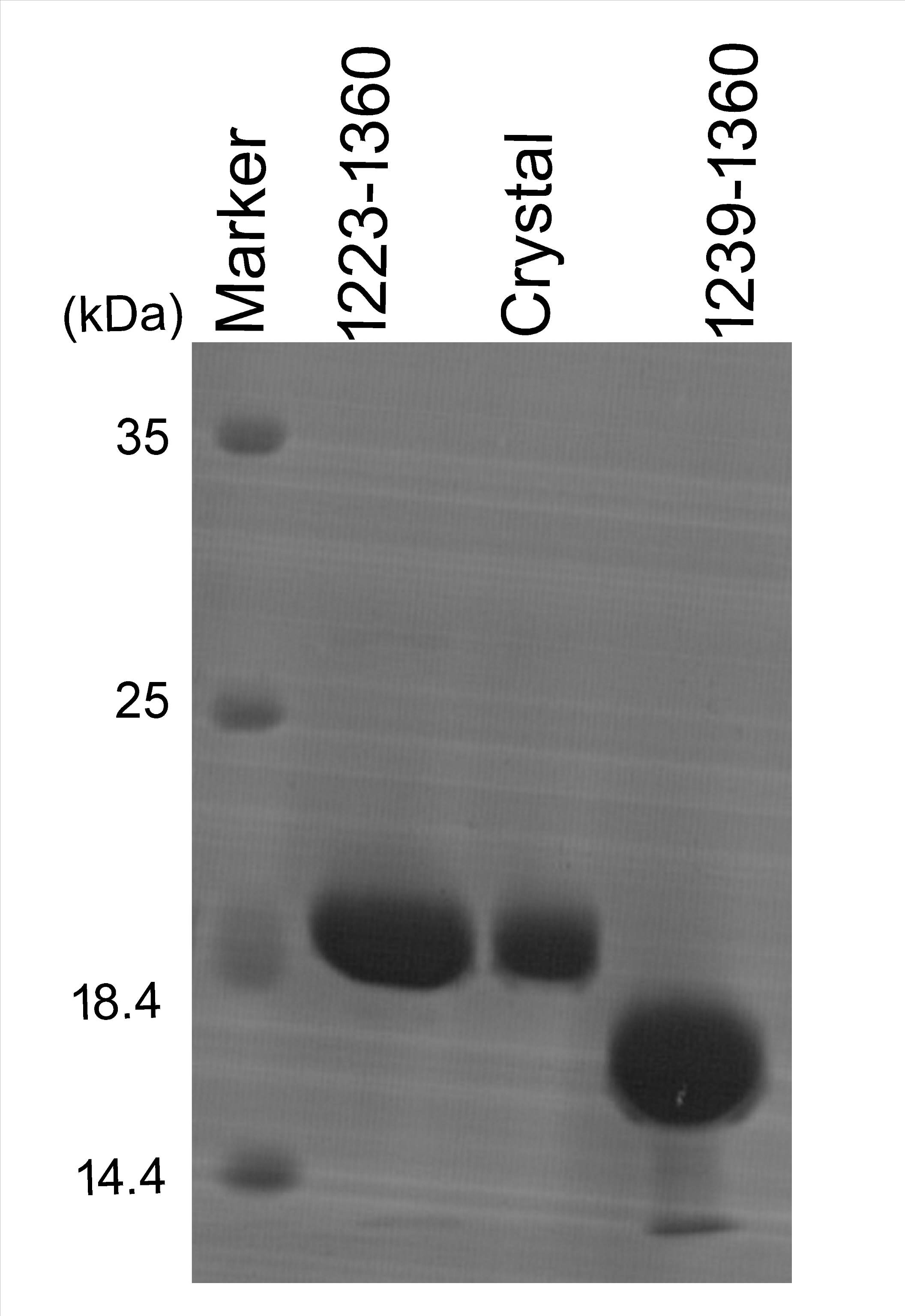


**Figure S2**. **The proteins before and after crystallization**

SDS-PAGE showing the purified REF6 fragment 1223-1360 and its crystals, along with the purified fragment 1239-1360. The crystals were washed 4 times with mother liquor.


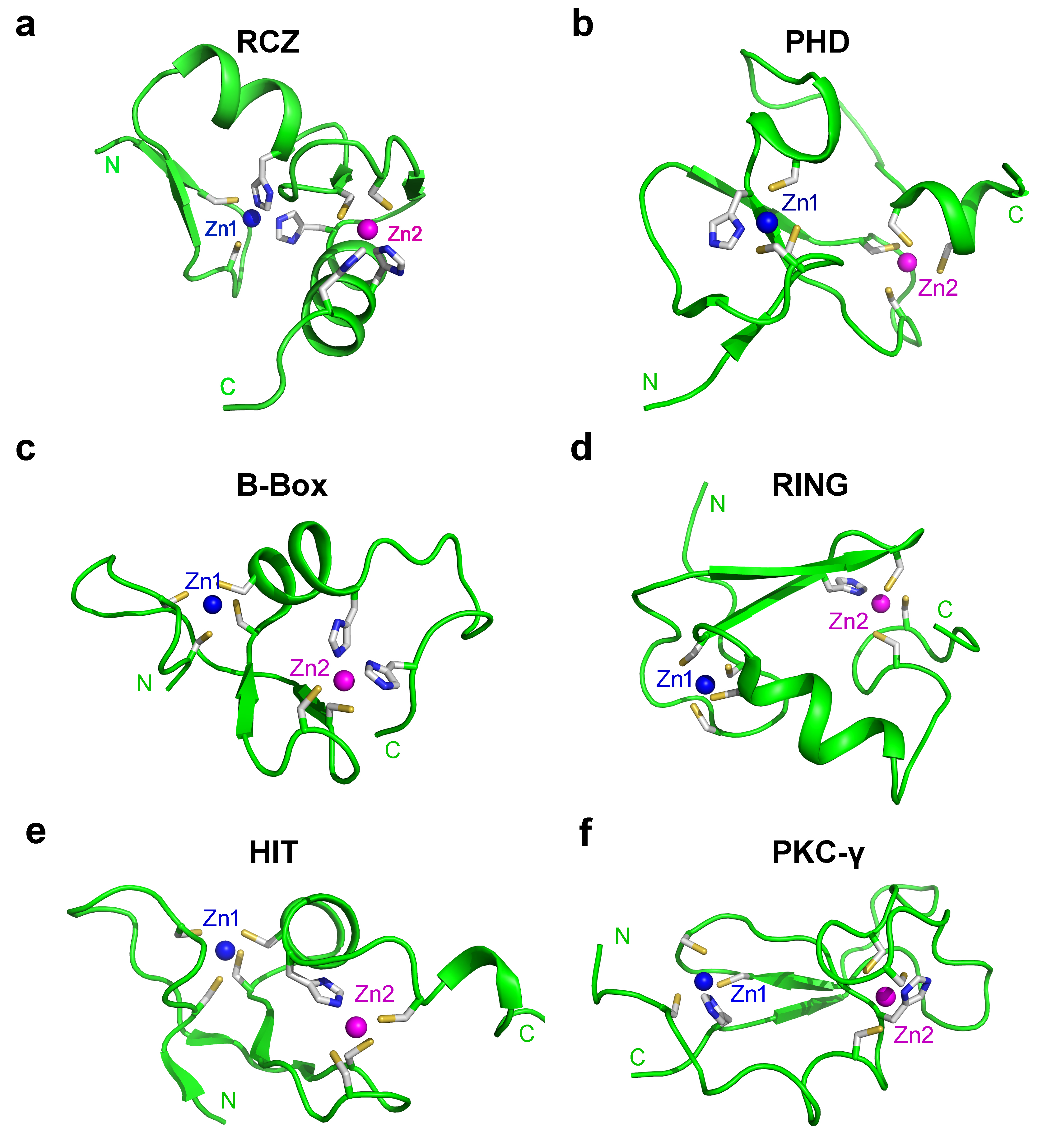


**Figure S3. Ribbon representation of the cross-braced ZnFs**

The structures of the cross-braced ZnFs, (a) RCZ (this study), (b) PHD (2G6Q[^2^](#_ENREF_2)), (c) B-Box (2FFW[^3^](#_ENREF_3)), (d) RING (1CHC[^4^](#_ENREF_4)), (e) HIT (1X4S[^5^](#_ENREF_5)), and (f) PKC-γ (1PTQ[^6^](#_ENREF_6)). The Zn1 and Zn2 atoms are shown as blue and magenta spheres, respectively. The main chains are colored green and the side chains of the coordinating residues are shown as sticks.


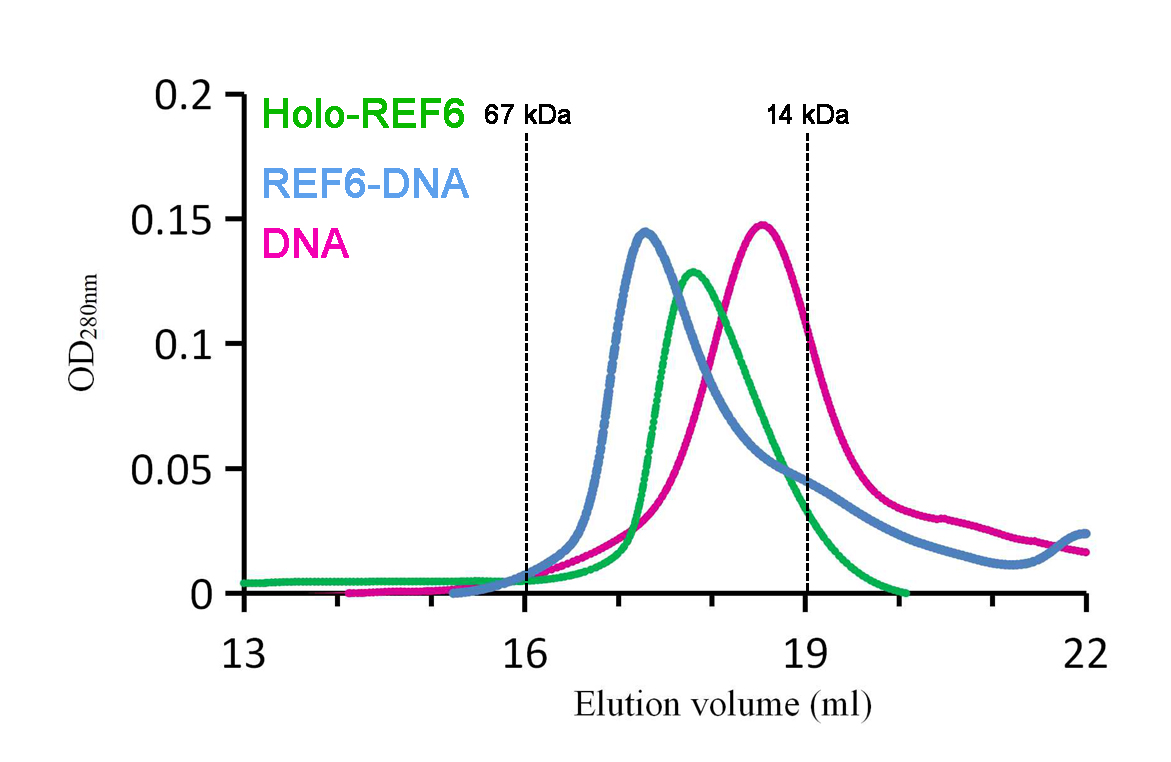


**Figure S4. Analytical gel filtration profiles of holo-REF6 and REF6-DNA complex at 2 mg ml^-1^**

REF6^1223-1360^ and the *NAC004* DNA fragment are used here. Elution volumes of the protein standards on a Superdex 200 10/300 GL column are marked at the top of the figure, respectively.


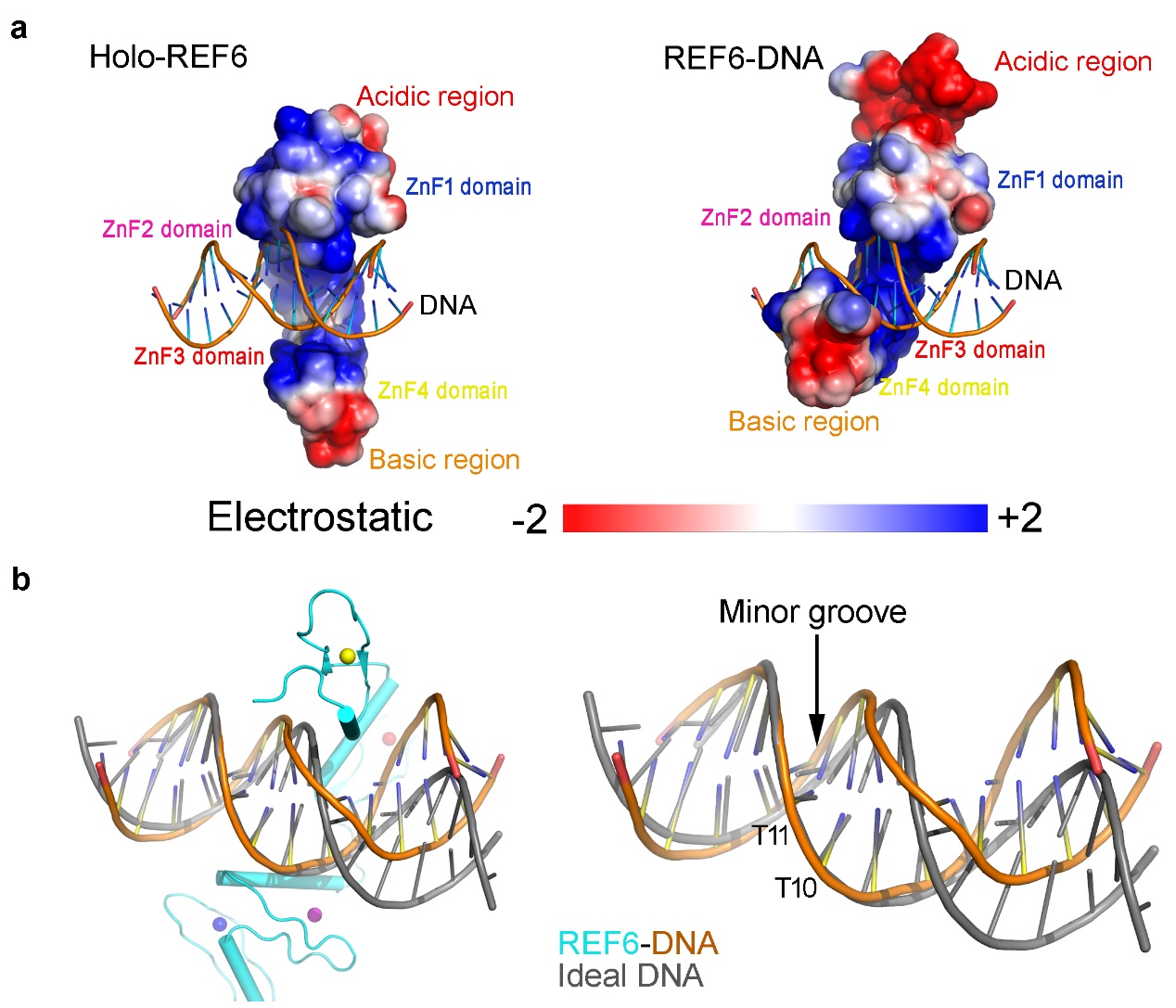


**Figure S5**. **Characteristics of holo-REF6 and** **REF6-DNA**

(a) The electrostatic potential (±2kBT) of holo-REF6 (left) and REF6-DNA complex (right). The surface potential is displayed as a color gradient ranging from red (negative) to blue (positive). Note that the dsDNA in the holo-REF6 is from the superposition of the REF6-DNA complex. (b) The overlay of the bound DNA duplex with a standard B-form DNA duplex, highlighting the differences in the minor groove. The DNA is enlarged in the right panel.

**a**


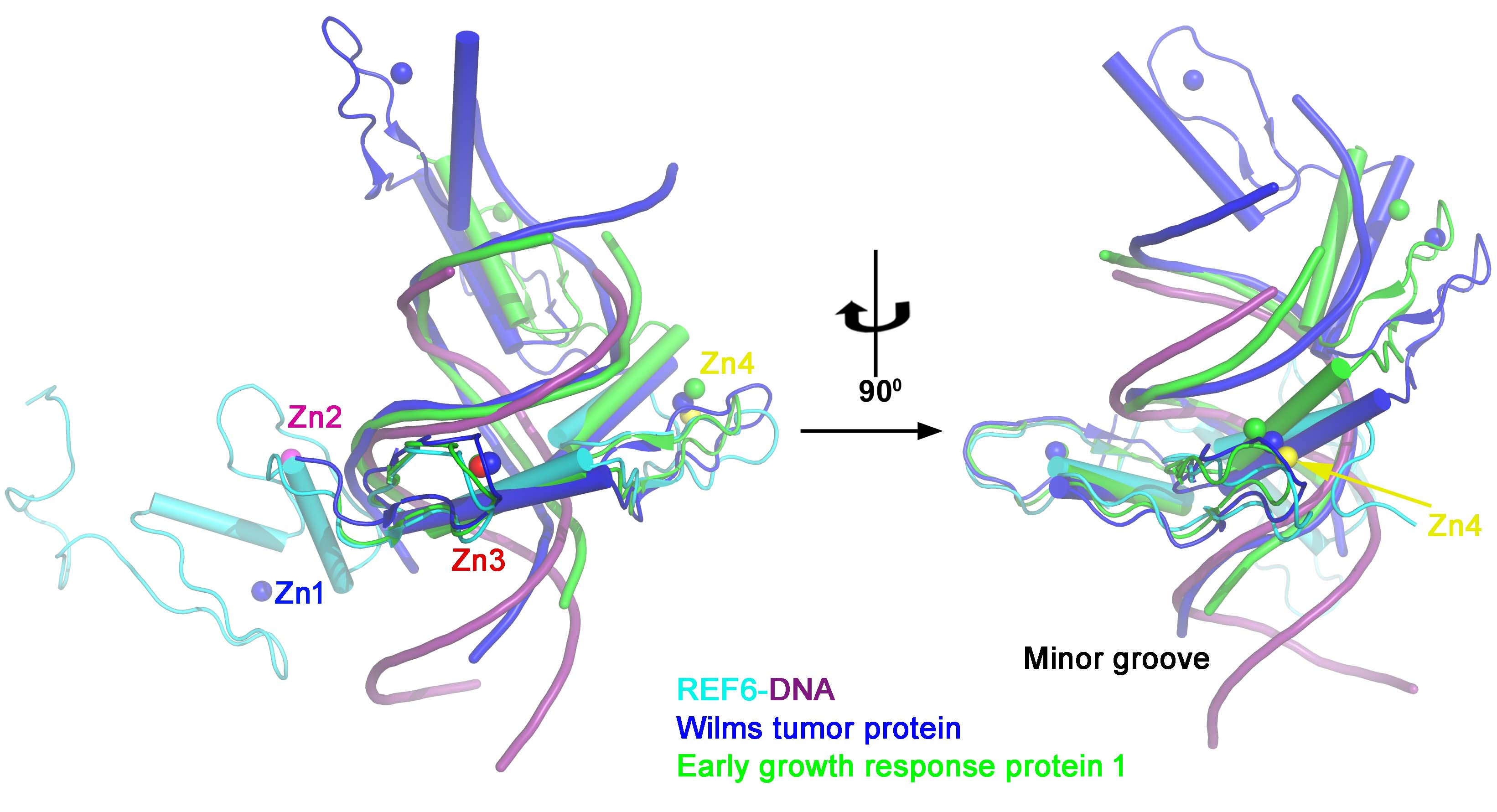


**b**


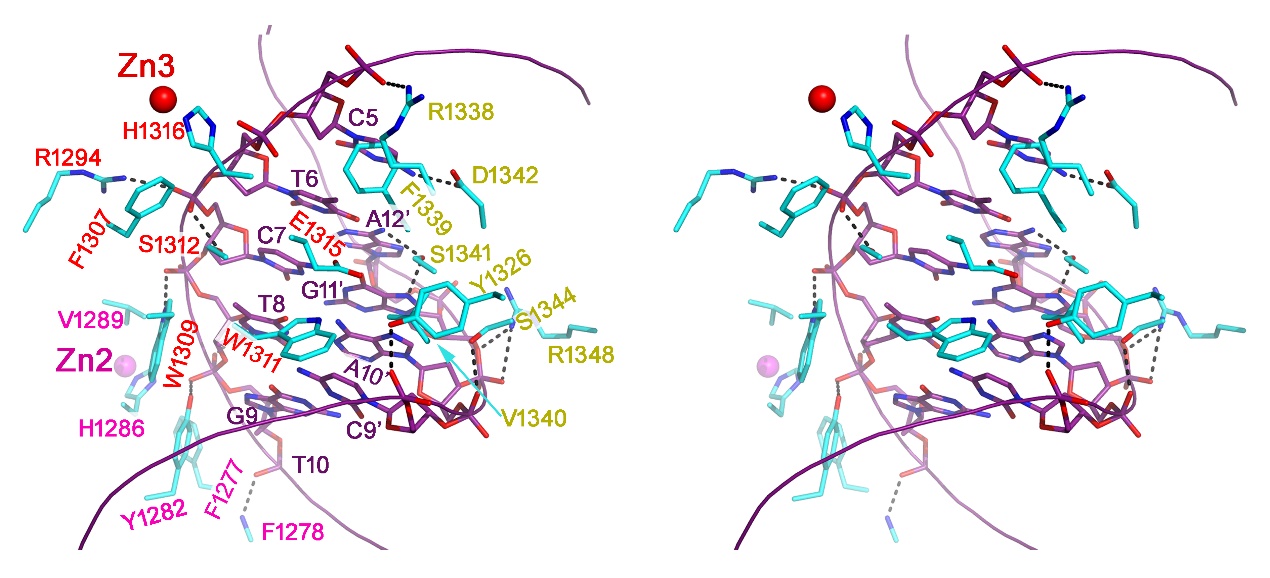


**Figure S6. Analyses of REF6-DNA complex**

(a) Overlay of the ZnF3-4 domain in the REF6-DNA complex (REF6 in cyan and DNA in purple) with the closest structural homolog, the ZnF1-2 domain of Wilms tumor protein (PDB: 6B0R, blue)[^7^](#_ENREF_7) and the ZnF1-2 domain of Early growth response protein 1 (PDB: 1A1F, green)[^8^](#_ENREF_8). Note that a 90° rotation view is shown in the right panel. Only two ZnF domains of REF6 directly insert into the DNA major groove, compared with 3 or 4 ZnF domains in the closest structural homologs. The sequence similarity between REF6 and these two proteins is less than 30%. In addition, no minor groove geometry was found for these two proteins as calculated by CURVES+[^9^](#_ENREF_9). (b) The sequence of the dsDNA used for crystallization is shown with two complementary strands. The residues involved in the interaction with the dsDNA are colored magenta, red, and yellow for domains ZnF2, 3, and 4, respectively. Solid and dashed arrows indicate hydrophobic and hydrophilic interactions, respectively.


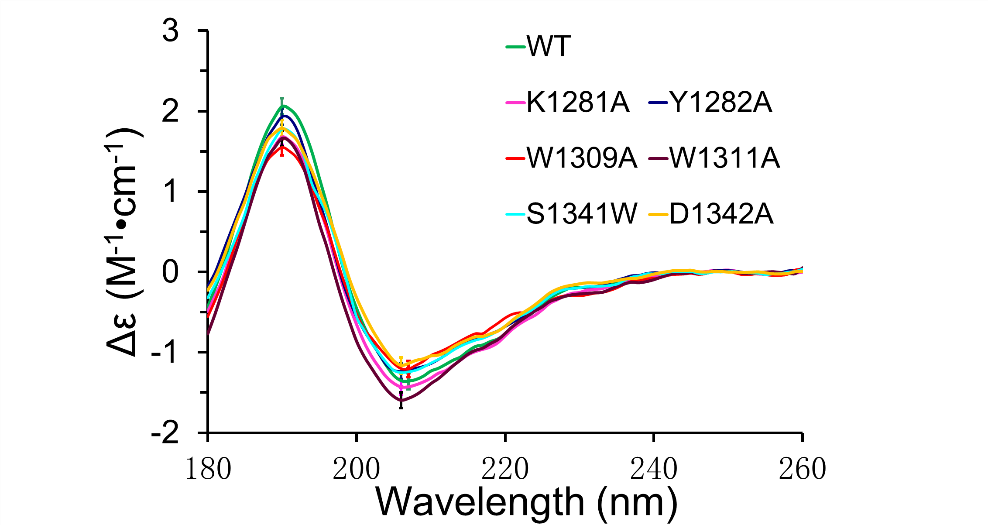


**Figure S7. Behaviors of REF6 and mutants**

CD spectra of the WT and the six mutants. The spectral shapes of WT and mutated REF6 were almost identical, indicating that the secondary structures of REF6 mutants were similar to that of WT.


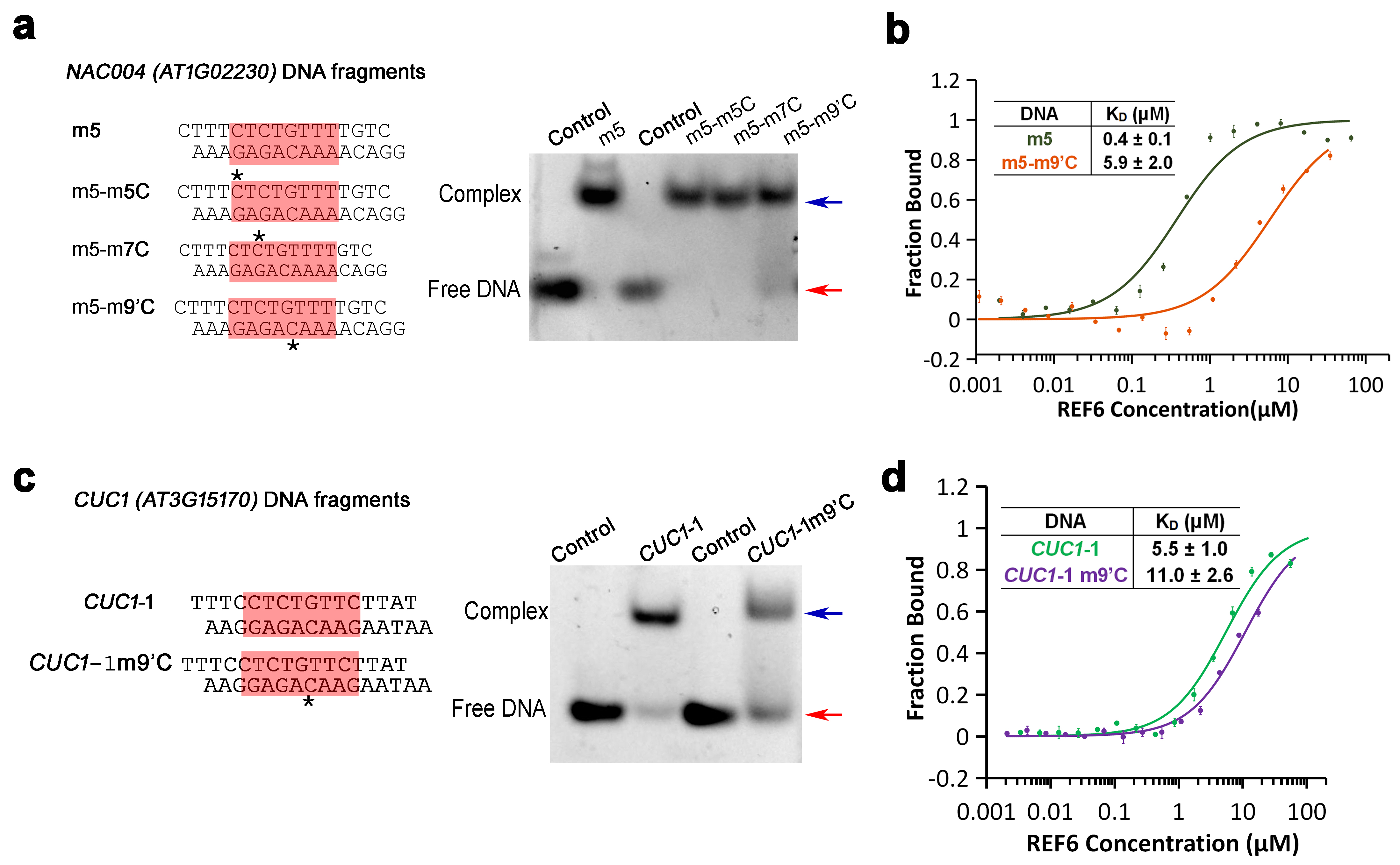


**Figure S8.** [**The influence of DNA methylation**](https://www.sogou.com/link?url=DSOYnZeCC_omHMBbSFWyQfrkPlX0eb3uitTRW3VK-hi1sBP6iSUmknwkqKQHKNUiYi7JNbl5jVHbSjkecD3bTabF6BBYbbki) **on recognition by REF6**

(a) EMSA of REF6 with the *NAC004* fragment. Methylation sites are indicated by an asterisk. (b) Comparison of DNA-binding affinities between m5 and m5-m9’C (methylation of cytosine base in 9’C). (c) EMSA of REF6 with the *CUC1*-1 fragments. (d) The DNA-binding affinity of *CUC1*-1 m9’C (methylation of cytosine base in 9’C) is lower than *CUC1*-1.


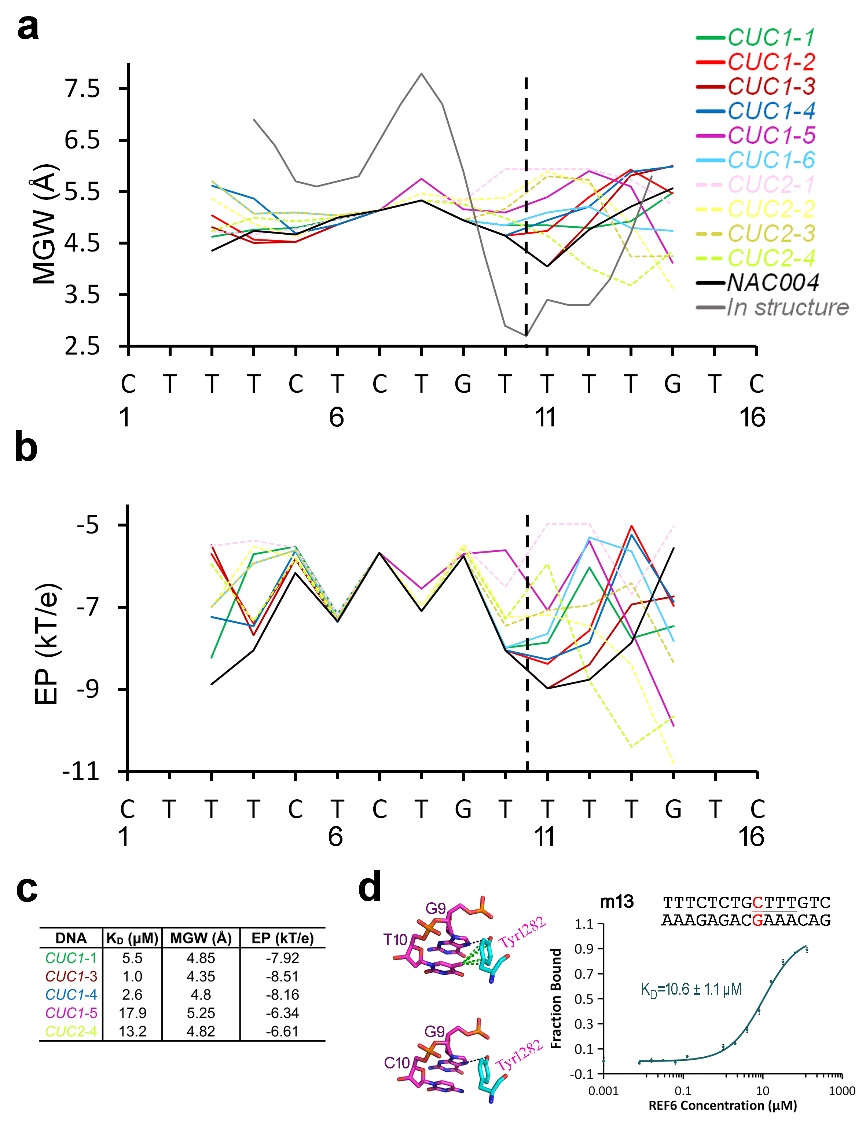


**Figure S9. The minor groove geometry and electrostatic potential of dsDNA**

Minor groove geometry (a) and electrostatic potential (b) of the potential DNA binding motifs in *CUC1*, *CUC2,* and *NAC004*. The minor groove width of the *NAC0004* in the complex structure is calculated by CURVES+[^9^](#_ENREF_9) and shown in the grey line. All the four *CUC2* sequences are shown as dashed lines. (c) Summary of tested K*_D_*, and predicted MGW and EP for selected motifs in the middle of bases T10 and T11*.* (d) Modeling of the m13 (mutated T:A base pair with a C:G base pair) and REF6 interaction. The mutation from T10 to C10 reduces the hydrophobic interactions with REF6. MST measurement of the binding affinity of m13 with REF6.


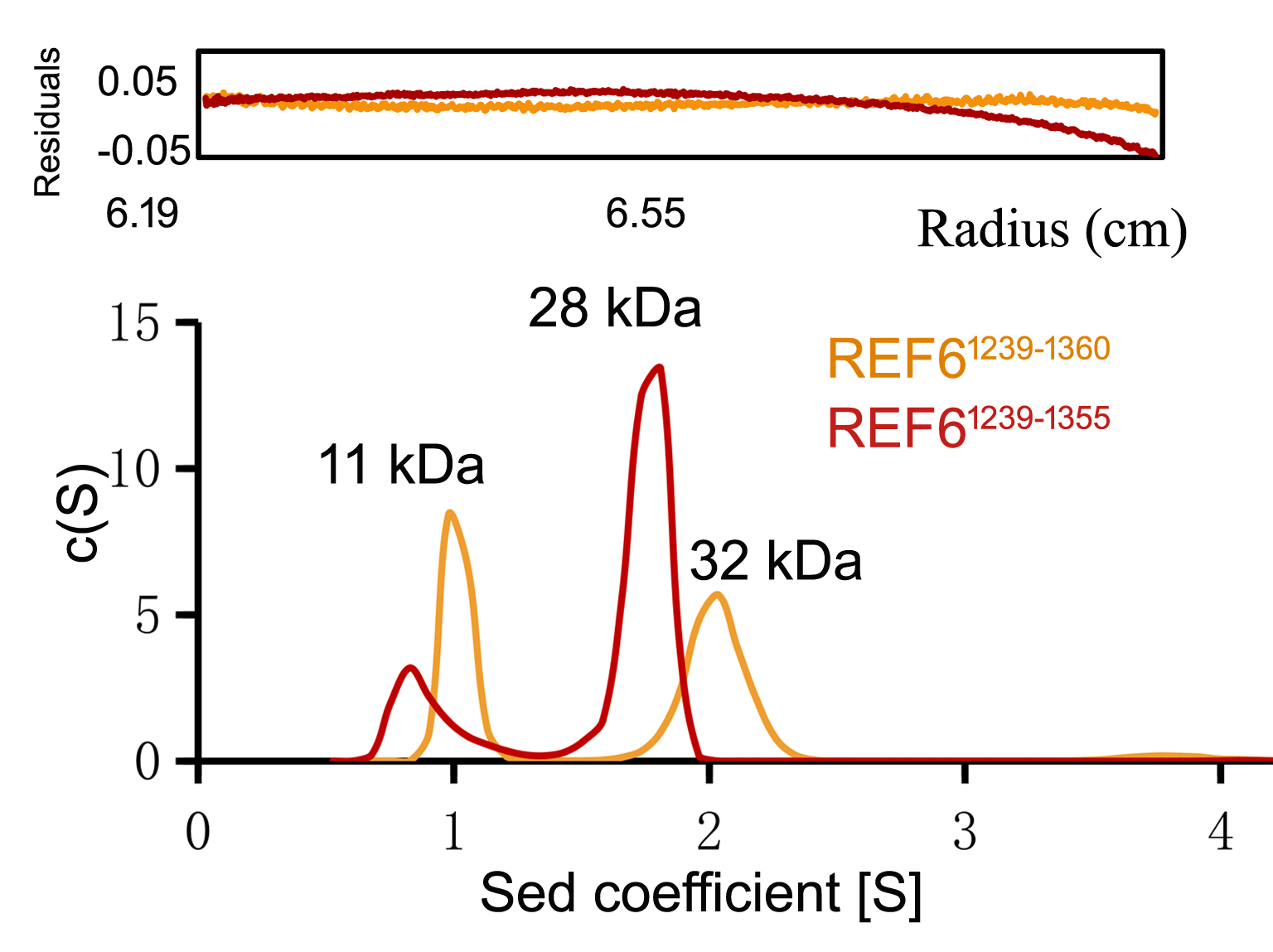


**Figure S10. The self-association of REF6 by** **sedimentation velocity (SV) AUC analysis**

The oligomeric state of REF6-DNA complex, including fragments 1239–1355 and 1239–1360, by analytical ultracentrifugation assays at 1.0 mg ml^-1^. The c(*s*) distribution from SV analysis is shown.


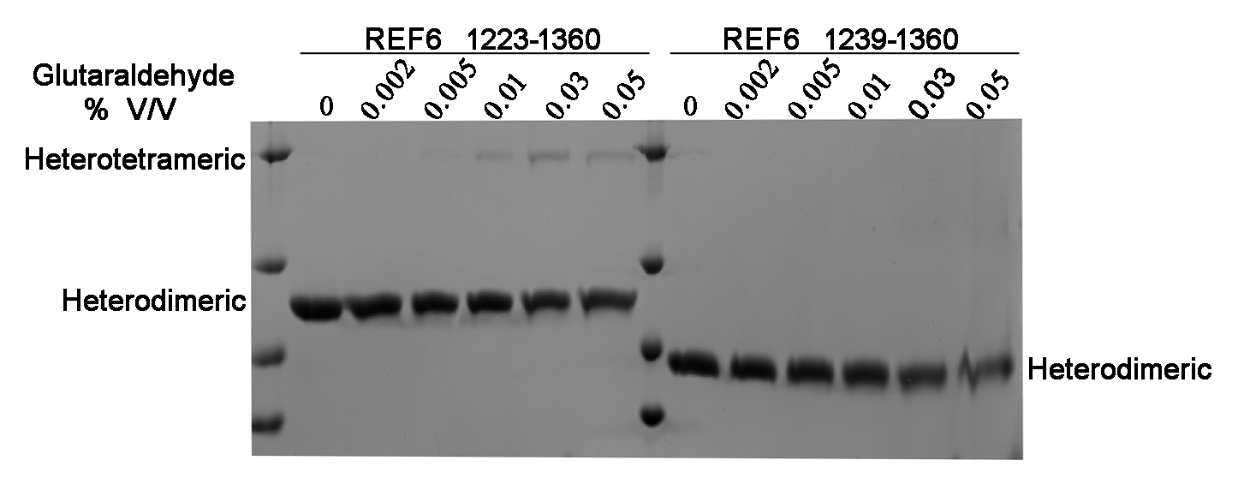


**Figure S11. Chemical crosslinking showed REF6^1223-1360^ existed in heterotetrameric state**

The fragment of REF6^1223-1360^ and REF6^1239-1360^ at 0.15 mg ml^-1^ were subjected to crosslinking reaction under different concentrations of crosslinking reagent glutaraldehyde.


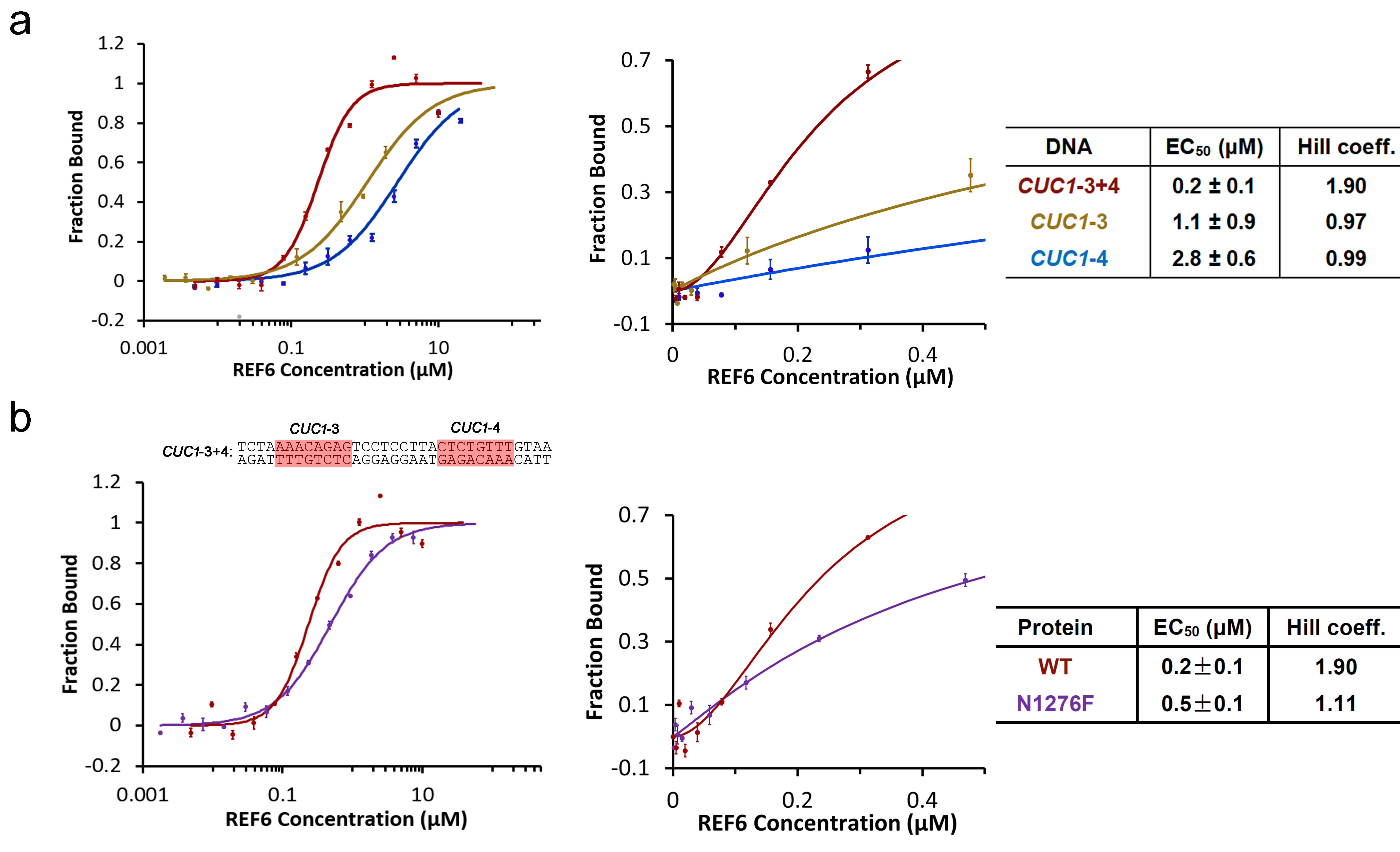


**Figure S12. The cooperativity of REF6 binding to dsDNA**

The range of 0-0.5 μM is enlarged at the right panel. (a) The Hill coefficient of *CUC1*-3+4 is larger than 1, indicating the existence of positive cooperativity. Comparison of the Hill coefficient of *CUC1*-3, *CUC1*-4, and *CUC1*-3+4 with REF6^1223-1360^. (b) The mutated residue N1276F obviously decreased the Hill coefficient of REF6^1223-1360^ for the *CUC1*-3+4. The experiments were repeated three times.


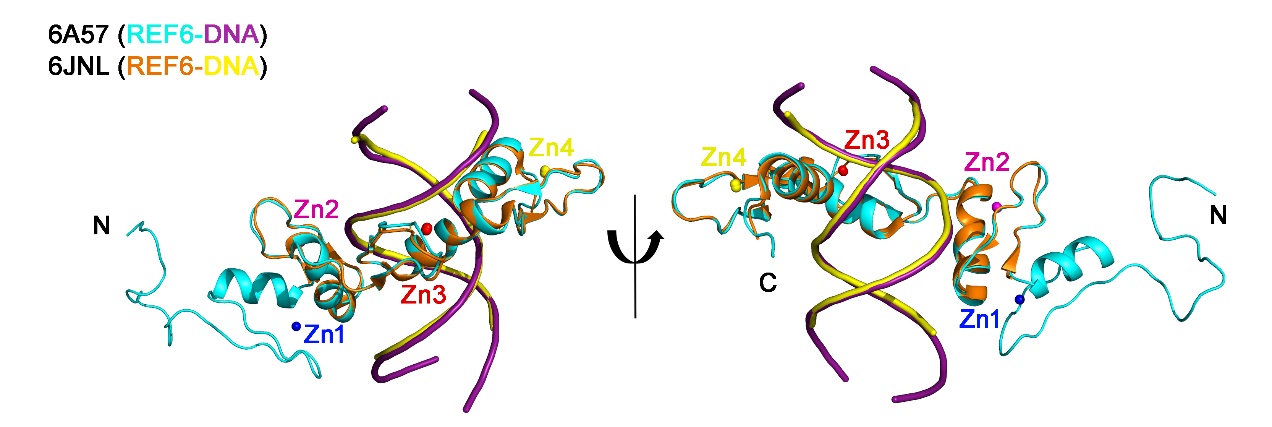


**Figure S13. Structural overlay between the REF6-DNA complexes**

The complex structures of REF6 ZnF2-4 (6JNL[^10^](#_ENREF_10)) and ours (6A57) had similar conformation. However, the sequence lengths of protein and nucleic acid in this study are significantly longer.

**Table S1.** Data collection and refinement statistics of holo-REF6 and REF6-DNA complex

|  | Holo-REF6 | |  | REF6-DNA complex |
| --- | --- | --- | --- | --- |
|  | Anomalous | | 6A58 |  |
| **Data collection** |  | |  |  |
| Wavelength | 0.97876 | | 1.28357 | 0.9793 |
| Space group | *P*4_1_ | | *P*4_1_ | *P*3_2_21 |
| Cell dimensions |  | |  |  |
| a,b,c (Å) | 63.63, 63.63, 43.62 | | 62.49, 62.49, 43.16 | 96.09, 96.09, 73.91 |
| α,β,γ (˚) | 90, 90, 90 | | 90, 90, 90 | 90, 90, 120 |
| Resolution (Å) | 50.0-2.05 (2.09-2.05) | | 50.0-1.57 (1.60-1.57) | 50.0-2.70 (2.75-2.70) |
| *R*_sym_ (%) | 6.5 | | 5.7 | 5.2 |
| *I*/σ | 29.0 (2.9) | | 76.8 (2.1) | 39.5 (2.2) |
| Completeness (%) | 99.9 (100) | | 99.7 (99.1) | 99.7 (96.0) |
| Total No. of reflections | 187409 | | 985699 | 375795 |
| Unique reflections | 11122 | | 23425 | 11190 |
| Redundancy | 6.8 (6.8) | | 12.8 (7.0) | 9.1 (6.0) |
| **Refinement** |  | |  |  |
| Resolution (Å) |  | | 50.0-1.57 (1.61-1.57) | 50.0-2.70 (2.75-2.70) |
| No. of reflections |  | | 22150 (1656) | 10516 (717) |
| *R*_work_/*R*_free_ (%) |  | | 21.1/23.4 | 22.1/25.7 |
| No. of atoms |  | |  |  |
| Protein |  | | 948 | 1001 |
| DNA |  | |  | 650 |
| Ligand/ions |  | | 4 | 16 |
| Water |  | | 119 | 79 |
| Average *B*-factors (Å^2^) |  | |  |  |
| Protein |  | | 36.51 | 60.63 |
| DNA |  | |  | 39.70 |
| Ligand/ion |  | | 39.64 | 44.08 |
| Water |  | | 46.29 | 39.67 |
| rms deviations |  | |  |  |
| Bond lengths (Å) |  | | 0.006 | 0.006 |
| Bond angles (º) |  | | 0.967 | 1.099 |
| Ramachandran Plot (%)^1^ | |  | 97/3/0 | 88/10/2 |

*R_sym_=*Σ*_h_*Σ*_i_|I_h,i_-I_h_|/*Σ*_h_*Σ*_l_I_h,i,_* where *I_h_* is the mean intensity of the *i^th^* observations of symmetry-related reflections of *h*.

^1^Residues in favored, allowed, and outlier regions of the Ramachandran plot.

**Table S2.** Summary of the residue-specific interactions between REF6 and the nucleotides of the DNA fragment (m5).

| Number | Nucleotide | Interaction with base | Interaction with DNA backbone | Residues | ZnF domain |
| --- | --- | --- | --- | --- | --- |
| 1 | C |  |  |  |  |
| 2 | T |  | √ | 1349 | ZnF4 |
| 3 | T |  | √ | 1345 | ZnF4 |
| 4 | T |  |  |  |  |
| 5 | C | √ |  | 1342 1339 | ZnF4 |
|  |  |  | √ | 1338 | ZnF4 |
|  |  | √ |  | 1309 | ZnF3 |
| 6 | T | √ |  | 1339 1341 | ZnF4 |
|  |  |  | √ | 1316 | ZnF3 |
| 7 | C | √ |  | 1341 | ZnF4 |
|  |  |  | √ | 1348 | ZnF4 |
|  |  |  | √ | 1312, 1294, 1307 | ZnF3 |
|  |  | √ |  | 1315 | ZnF3 |
| 8 | T | √ |  | 1311 | ZnF3 |
|  |  |  | √ | 1347 1344 | ZnF4 |
|  |  | √ | √ | 1340 | ZnF4 |
|  |  |  | √ | 1309 | ZnF3 |
| 9 | G |  | √ | 1326 | ZnF4 |
|  |  | √ |  | 1311 | ZnF3 |
|  |  | √ | √ | 1282 | ZnF2 |
|  |  |  | √ | 1275, 1286 | ZnF2 |
| 10 | T | √ |  | 1311 | ZnF3 |
|  |  | √ |  | 1282 | ZnF2 |
|  |  |  | √ | 1278 | ZnF2 |
| 11 | T |  |  |  |  |
| 12 | T |  |  |  |  |
| 13 | T |  |  |  |  |
| 14 | G |  |  |  |  |
| 15 | T |  |  |  |  |
| 16 | C |  |  |  |  |

Note: The residues involved in the interaction with the dsDNA are colored magenta, red, and yellow for domains ZnF2, 3, and 4, respectively.

**Table S3.** SAXS results for holo-REF6 and REF6-DNA complex.

(a) Sample details.

| Protein | Holo-REF6^1223-1360^ | Holo-REF6^1239-1360^ | | REF6^1223-1360^-DNA complex | | REF6^1239-1360^-DNA complex | |
| --- | --- | --- | --- | --- | --- | --- | --- |
| Organism | Arabidopsis thaliana | | Arabidopsis thaliana | | Arabidopsis thaliana | | Arabidopsis thaliana |
| Source | E. coli  expressed | | E. coli expressed | | E. coli expressed | | E. coli  expressed |
| UniProt sequence ID  (residues in construct) | Q9STM3  (1223–1360) | | Q9STM3 (1239–1360) | | Q9STM3 (1223–1360) | | Q9STM3 (1239–1360) |
| Extinction coefficient [A_280_, 0.1%(w/v)] | 1.314 | | 1.499 | |  | |  |
| Molecular mass *M* from chemical composition (kDa) | 16.34 | | 14.32 | | 26.10 | | 24.08 |
| concentration (mg ml^−1^) | 5.0 | | 5.0 | | 5.0 | | 5.0 |
| $\overline{\upsilon}$ from chemical composition (cm^3^ g^−1^) | 0.719 | | 0.726 | | 0.671 | | 0.672 |
| Particle contrast from sequence and solvent constituents, $\Delta\overline{\rho}$ (ρ_protein_ − ρ_solvent_; 10^10^ cm^−2^) | 0.84 (12.57-11.73) | | 2.91 (12.47-9.56) | | 3.86 (13.32-9.46) | | 3.85 (13.31- 9.46) |
| Solvent | 20 mM Hepes pH 7.5  500 mM NaCl | | 20 mM Hepes pH 7.5  500 mM NaCl | | 25 mM MES pH 6.0  150 mM NaCl, | | 25 mM MES pH 6.0  150 mM NaCl, |

(b) SAXS data-collection parameters.

| Instrument/data processing | SSRF BL19U2 with Dectris PILATUS 1M detector | | | |
| --- | --- | --- | --- | --- |
| Wavelength (Å) | 1.03 | | | |
| Beam size (µm) | 320 × 43 | | | |
| Camera length (m) | 2.68 | | | |
| *q*-measurement range (Å^−1^) | 0.007-0.355 |  |  |  |
| Absolute scaling method | Comparison with scattering from 1 mm pure H_2_O | | | |
| Normalization | To transmitted intensity by beam-stop counter | | | |
| Monitoring for radiation damage | X-ray dose maintained below 210 Gy, data frame-by-frame comparison | | | |
| Exposure time | Continuous 1 s data-frame measurements | | | |
| Sample temperature (°C) | 10.0 | | | |

(c) Software employed for SAXS data reduction, analysis and interpretation.

| SAXS data reduction | RAW[^11^](#_ENREF_11) |
| --- | --- |
| Extinction coefficient estimate | ProtParam (Gasteiger et al., 2005) |
| Calculation of $\Delta\overline{\rho}$ and $\overline{\upsilon}$ values | MULCh 1.1[^12^](#_ENREF_12) |
| Basic analyses: Guinier, P(r), V_P_ | RAW[^11^](#_ENREF_11) |
| Atomic structure modelling | FoXS[^13^](#_ENREF_13) |

(d) Structural parameters.

| Protein | Holo-REF6^1223-1360^ | Holo-REF6^1239-1360^ | REF6^1223-1360^-DNA complex | REF6^1239-1360^-DNA complex |
| --- | --- | --- | --- | --- |
| Guinier analysis | | | | |
| *I*(0) (cm^−1^) | 47.91 | 28.16 | 18.67 | 5.99 |
| *R*_g_ (Å) | 28.21 | 22.77 | 25.98 | 20.46 |
| *q*_min_ (Å^-1^) | 0.0171 | 0.0223 | 0.0248 | 0.0292 |
| *qR*_g_ max | 1.30 | 1.30 | 1.30 | 1.30 |
| Coefficient of correlation, R^2^ | 0.992 | 0.987 | 0.988 | 0.967 |
| *M* from *I*(0) (ratio to predicted) | 29.2 (0.89)^1^ | 12.0 (0.84)^2^ | 31.8 (0.61)^3^ | 12.8 (0.53)^4^ |
| *P*(r) analysis |  | | | |
| *I*(0) (cm^−1^) | 47.76 | 28.44 | 18.85 | 5.99 |
| *R*_g_ (Å) | 28.82 | 23.77 | 26.89 | 20.77 |
| *q*-range (Å^-1^) | 0.018-0.30 | 0.018-0.30 | 0.015-0.30 | 0.015-0.30 |
| *d*_max_ (Å) | 95 | 80 | 91 | 71 |
| χ^2^ (total estimate from GNOM) | 0.624 (0.814) | 0.643 (0.859) | 0.697 (0.892) | 0.665 (0.933) |
| *M* from *I*(0) (ratio to predicted value) | 24.0 (0.73)^1^ | 12.2 (0.84)^2^ | 31.7 (0.61)^3^ | 13.2 (0.55)^4^ |
| Porod volume *V*p (Å^−3^) | 50974.1 | 30210.4 | 64439.7 | 31773.0 |
| Correlation volume *V*c (Å^−2^) | 283.5 | 186.4 | 302.5 | 188.1 |

(e) Atomistic modelling.

| Protein | Holo-REF6^1223-1360^ | Holo-REF6^1239-1360^ | REF6^1223-1360^-DNA complex | REF6^1239-1360^-DNA complex |
| --- | --- | --- | --- | --- |
| Crystal structure | Symmetric operation based on the crystal structure (PDB entry 6A58) | PDB entry 6A58 | Symmetric operation based on the crystal structure (PDB entry 6A57) | PDB entry 6A57 |
| *q-*range (Å^-1^) | 0.010-0.300 | 0.010-0.300 | 0.007-0.300 | 0.007-0.300 |
| *FoXS* |  | | | |
| χ^2^ | 5.51 | 2.38 | 5.47 | 3.69 |
| Predicted R_g_ (Å) | 25.43 | 21.18 | 23.41 | 18.34 |
| c_1_, c_2_ | 0.99, -0.39 | 0.99, 2.33 | 1.05, -2.00 | 1.05, -0.25 |

In FoXS, the adjustable parameters c_1_ and c_2_ are adjustments for excluded volume and hydration density. c_1_ can vary by 5% (0.95–1.05). c_2_ is allowed to be slightly negative (-2 ≤ c_2_ ≤ 4). The maximum hydration adjustment c_2_ of 4.0 corresponds to ∼0.388 e Å^−3^ (compared with bulk solvent density ρ = 0.334 e Å^−3^) and the minimum hydration adjustment c_2_ of -2.0 corresponds to ∼0.307 e Å^−3^.

^1^ In Guinier analysis and *P*(r) analysis, it is the ratio of the calculated value to the predicted molecular mass of Holo-REF6^1223-1360^ dimer.

^2^ In Guinier analysis and *P*(r) analysis, it is the ratio of the calculated value to the predicted molecular mass of Holo-REF6^1239-1360^ monomer.

^3^ In Guinier analysis and *P*(r) analysis, it is the ratio of the calculated value to the predicted molecular mass of Heterotetrameric REF6^1223-1360^-DNA complex.

^4^ In Guinier analysis and *P*(r) analysis, it is the ratio of the calculated value to the predicted molecular mass of Heterodimeric REF6^1239-1360^-DNA complex.

**Movie S1.**

The complex structure of the REF6-DNA complex (REF6 in cyan and DNA in purple) structure. The four zinc ions are presented as blue, magenta, red, and yellow spheres, respectively.

**Supplementary References**

1 Sheldrick, G. M. A short history of SHELX. *Acta Crystallogr. A* **64**, 112-122 (2008).

2 Pena, P. V. *et al.* Molecular mechanism of histone H3K4me3 recognition by plant homeodomain of ING2. *Nature* **442**, 100-103 (2006).

3 Garcia-Saez, I., Tcherniuk, S. & Kozielski, F. The structure of human neuronal Rab6B in the active and inactive form. *Acta Crystallogr. D Biol. Crystallogr.* **62**, 725-733 (2006).

4 Barlow, P. N., Luisi, B., Milner, A., Elliott, M. & Everett, R. Structure of the C3HC4 domain by 1H-nuclear magnetic resonance spectroscopy. A new structural class of zinc-finger. *J. Mol. Biol.* **237**, 201-211 (1994).

5 He, F. *et al.* Solution structure of the zinc finger HIT domain in protein FON. *Protein Sci.* **16**, 1577-1587 (2007).

6 Zhang, G., Kazanietz, M. G., Blumberg, P. M. & Hurley, J. H. Crystal structure of the cys2 activator-binding domain of protein kinase C delta in complex with phorbol ester. *Cell* **81**, 917-924 (1995).

7 Wang, D. *et al.* Role for first zinc finger of WT1 in DNA sequence specificity: Denys-Drash syndrome-associated WT1 mutant in ZF1 enhances affinity for a subset of WT1 binding sites. *Nucleic Acids Res.* **46**, 3864-3877 (2018).

8 Elrod-Erickson, M., Benson, T. E. & Pabo, C. O. High-resolution structures of variant Zif268-DNA complexes: implications for understanding zinc finger-DNA recognition. *Structure* **6**, 451-464 (1998).

9 Lavery, R., Moakher, M., Maddocks, J. H., Petkeviciute, D. & Zakrzewska, K. Conformational analysis of nucleic acids revisited: Curves+. *Nucleic Acids Res.* **37**, 5917-5929 (2009).

10 Qiu, Q. *et al.* DNA methylation repels targeting of Arabidopsis REF6. *Nat. Commun.* **10**, 2063 (2019).

11 Hopkins, J. B., Gillilan, R. E. & Skou, S. BioXTAS RAW: improvements to a free open-source program for small-angle X-ray scattering data reduction and analysis. *J. Appl. Crystallogr.* **50**, 1545-1553 (2017).

12 Whitten, A. E., Cai, S. & Trewhella, J. MULCh: modules for the analysis of small-angle neutron contrast variation data from biomolecular assemblies. *J. Appl. Crystallogr.* **41**, 222-226 (2008).

13 Schneidman-Duhovny, D., Hammel, M., Tainer, J. A. & Sali, A. Accurate SAXS profile computation and its assessment by contrast variation experiments. *Biophys. J.* **105**, 962-974 (2013).
