# Supplementary material for: Crystal structures of REF6 and its complex with DNA reveal diverse recognition mechanisms
Source: Cell Discov. 2020 Mar 31;6:17. doi: 10.1038/s41421-020-0150-6 (PMC7105484; doi:10.1038/s41421-020-0150-6)
Supplement: Supplementary file 2 — Validation report [file 41421_2020_150_MOESM2_ESM.pdf]

Full wwPDB X-ray Structure Validation Report (i)

Jun 26, 2018 – 02:37 PM JST

PDB ID	:	6A57
Title	:	Structure of histone demethylase REF6 complexed with DNA
Deposited on	:	2018-06-22
Resolution	:	2.70 Å(reported)

This is a Full wwPDB X-ray Structure Validation Report.

This report is produced by the wwPDB biocuration pipeline after annotation of the structure.

We welcome your comments at *validation@mail.wwpdb.org*
A user guide is available at
https://www.wwpdb.org/validation/2017/XrayValidationReportHelp
with specific help available everywhere you see the (i) symbol.

CONFIDENTIAL VALIDATION REPORT

The following versions of software and data (see references (i)) were used in the production of this report:

MolProbity	:	4.02b-467
Mogul	:	1.7.3 (157068), CSD as539be (2018)
Xtriage (Phenix)	:	1.13
EDS	:	rb-20031172
Percentile statistics	:	20171227.v01 (using entries in the PDB archive December 27th 2017)
Refmac	:	5.8.0158
CCP4	:	7.0 (Gargrove)
Ideal geometry (proteins)	:	Engh & Huber (2001)
Ideal geometry (DNA, RNA)	:	Parkinson et al. (1996)
Validation Pipeline (wwPDB-VP)	:	rb-20031172

1 Overall quality at a glance (i)

The following experimental techniques were used to determine the structure:
X-RAY DIFFRACTION

The reported resolution of this entry is 2.70 Å.

Percentile scores (ranging between 0-100) for global validation metrics of the entry are shown in the following graphic. The table shows the number of entries on which the scores are based.

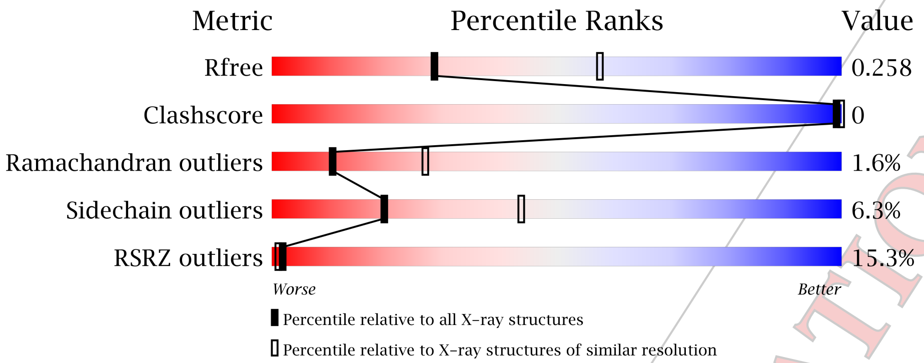

Metric	Whole archive (#Entries)	Similar resolution (#Entries, resolution range(Å))
R_{free}	111664	2449 (2.70-2.70)
Clashscore	122126	2756 (2.70-2.70)
Ramachandran outliers	120053	2716 (2.70-2.70)
Sidechain outliers	120020	2716 (2.70-2.70)
RSRZ outliers	108989	2376 (2.70-2.70)

The table below summarises the geometric issues observed across the polymeric chains and their fit to the electron density. The red, orange, yellow and green segments on the lower bar indicate the fraction of residues that contain outliers for >=3, 2, 1 and 0 types of geometric quality criteria. A grey segment represents the fraction of residues that are not modelled. The numeric value for each fraction is indicated below the corresponding segment, with a dot representing fractions <=5% The upper red bar (where present) indicates the fraction of residues that have poor fit to the electron density. The numeric value is given above the bar.

Mol	Chain	Length	Quality of chain
1	A	140	18% / 87% 6% • 6%
2	B	16	100%
3	C	16	100%

CONFIDENTIAL VALIDATION REPORT

2 Entry composition (i)

There are 6 unique types of molecules in this entry. The entry contains 1746 atoms, of which 0 are hydrogens and 0 are deuteriums.

In the tables below, the ZeroOcc column contains the number of atoms modelled with zero occupancy, the AltConf column contains the number of residues with at least one atom in alternate conformation and the Trace column contains the number of residues modelled with at most 2 atoms.

- Molecule 1 is a protein called Lysine-specific demethylase REF6.

Mol	Chain	Residues	Atoms					ZeroOcc	AltConf	Trace
			Total	C	N	O	S			
1	A	131	1001	622	193	176	10	0	0	0

There are 2 discrepancies between the modelled and reference sequences:

Chain	Residue	Modelled	Actual	Comment	Reference
A	1221	GLY	-	expression tag	UNP Q9STM3
A	1222	SER	-	expression tag	UNP Q9STM3

- Molecule 2 is a DNA chain called DNA (5'-D(*CP*TP*TP*TP*CP*TP*CP*TP*GP*TP*TP*TP*TP*GP*TP*C)-3').

Mol	Chain	Residues	Atoms					ZeroOcc	AltConf	Trace
			Total	C	N	O	P			
2	B	16	317	156	42	104	15	0	0	0

- Molecule 3 is a DNA chain called DNA (5'-D(*GP*GP*AP*CP*AP*AP*AP*AP*CP*AP*GP*AP*GP*AP*AP*A)-3').

Mol	Chain	Residues	Atoms					ZeroOcc	AltConf	Trace
			Total	C	N	O	P			
3	C	16	333	158	76	84	15	0	0	0

- Molecule 4 is ZINC ION (three-letter code: ZN) (formula: Zn).

Mol	Chain	Residues	Atoms		ZeroOcc	AltConf
			Total	Zn		
4	A	4	4	4	0	0

- Molecule 5 is GLYCEROL (three-letter code: GOL) (formula: $C_3H_8O_3$).

CONFIDENTIAL VALIDATION REPORT

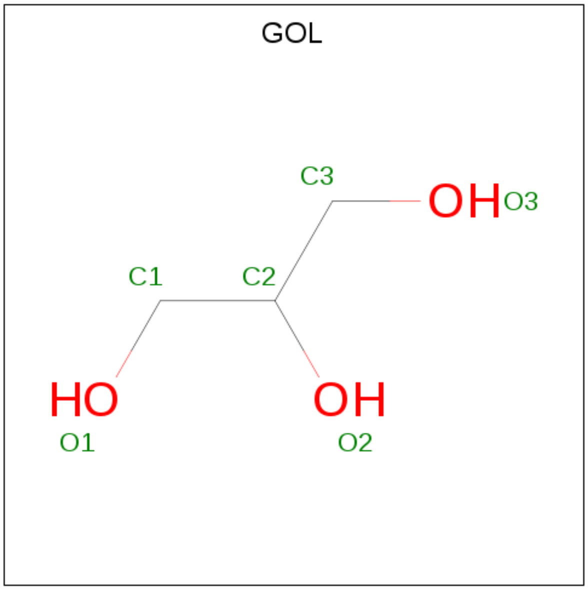

Mol	Chain	Residues	Atoms	ZeroOcc	AltConf
5	C	1	Total C O<br>6 3 3	0	0
5	C	1	Total C O<br>6 3 3	0	0

- Molecule 6 is water.

Mol	Chain	Residues	Atoms	ZeroOcc	AltConf
6	A	46	Total O<br>46 46	0	0
6	B	17	Total O<br>17 17	0	0
6	C	16	Total O<br>16 16	0	0

CONFIDENTIAL VALIDATION REPORT

3 Residue-property plots (i)

These plots are drawn for all protein, RNA and DNA chains in the entry. The first graphic for a chain summarises the proportions of the various outlier classes displayed in the second graphic. The second graphic shows the sequence view annotated by issues in geometryand electron density. Residues are color-coded according to the number of geometric quality criteria for which they contain at least one outlier: green = 0, yellow = 1, orange = 2 and red = 3 or more. A red dot above a residue indicates a poor fit to the electron density (RSRZ > 2). Stretches of 2 or more consecutive residues without any outlier are shown as a green connector. Residues present in the sample, but not in the model, are shown in grey.

• Molecule 1: Lysine-specific demethylase REF6

Chain A:

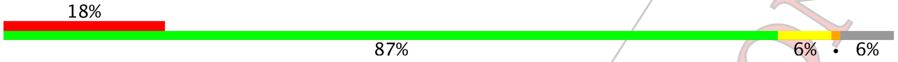

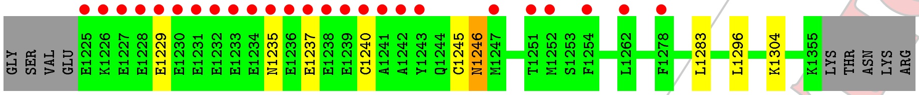

• Molecule 2: DNA (5'-D(*CP*TP*TP*TP*CP*TP*CP*TP*GP*TP*TP*TP*TP*GP*TP*C)-3 ')

Chain B:

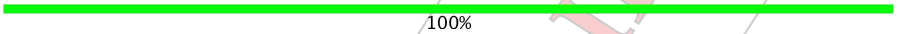

There are no outlier residues recorded for this chain.

• Molecule 3: DNA (5'-D(*GP*GP*AP*CP*AP*AP*AP*AP*CP*AP*GP*AP*GP*AP*AP*A)-3')

Chain C:

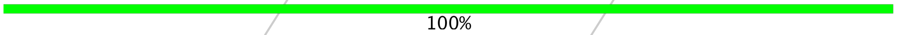

There are no outlier residues recorded for this chain.

CONFIDENTIAL VALIDATION REPORT

4 Data and refinement statistics (i)

Property	Value	Source
Space group	P 32 2 1	Depositor
Cell constants<br>a, b, c, α, β, γ	96.09Å 96.09Å 73.91Å<br>90.00° 90.00° 120.00°	Depositor
Resolution (Å)	50.01 – 2.70<br>48.04 – 2.70	Depositor<br>EDS
% Data completeness<br>(in resolution range)	99.5 (50.01-2.70)<br>99.5 (48.04-2.70)	Depositor<br>EDS
R_{merge}	(Not available)	Depositor
R_{sym}	(Not available)	Depositor
$< I/\sigma(I) >$ [1]	2.03 (at 2.69Å)	Xtriage
Refinement program	REFMAC 5.8.0158	Depositor
R, R_{free}	0.221 , 0.257<br>0.225 , 0.258	Depositor<br>DCC
R_{free} test set	596 reflections (5.36%)	wwPDB-VP
Wilson B-factor (Å^2)	46.4	Xtriage
Anisotropy	0.045	Xtriage
Bulk solvent k_{sol}(e/Å^3), B_{sol}(Å^2)	0.32 , 42.7	EDS
L-test for twinning[2]	$< \|L\| > = 0.50$, $< L^2 > = 0.34$	Xtriage
Estimated twinning fraction	0.036 for -h,-k,l	Xtriage
F_o,F_c correlation	0.92	EDS
Total number of atoms	1746	wwPDB-VP
Average B, all atoms (Å^2)	51.0	wwPDB-VP

Xtriage's analysis on translational NCS is as follows: *The largest off-origin peak in the Patterson function is 5.85% of the height of the origin peak. No significant pseudotranslation is detected.*

[1] Intensities estimated from amplitudes.

[2] Theoretical values of $< |L| >$, $< L^2 >$ for acentric reflections are 0.5, 0.333 respectively for untwinned datasets, and 0.375, 0.2 for perfectly twinned datasets.

CONFIDENTIAL VALIDATION REPORT

5 Model quality (i)

5.1 Standard geometry (i)

Bond lengths and bond angles in the following residue types are not validated in this section: GOL, ZN

The Z score for a bond length (or angle) is the number of standard deviations the observed value is removed from the expected value. A bond length (or angle) with $|Z| > 5$ is considered an outlier worth inspection. RMSZ is the root-mean-square of all Z scores of the bond lengths (or angles).

Mol	Chain	Bond lengths		Bond angles	
		RMSZ	$\#\|Z\| > 5$	RMSZ	$\#\|Z\| > 5$
1	A	0.40	0/1028	0.60	0/1388
2	B	0.34	0/350	0.79	0/538
3	C	0.34	0/378	0.78	0/582
All	All	0.37	0/1756	0.69	0/2508

There are no bond length outliers.

There are no bond angle outliers.

There are no chirality outliers.

There are no planarity outliers.

5.2 Too-close contacts (i)

In the following table, the Non-H and H(model) columns list the number of non-hydrogen atoms and hydrogen atoms in the chain respectively. The H(added) column lists the number of hydrogen atoms added and optimized by MolProbity. The Clashes column lists the number of clashes within the asymmetric unit, whereas Symm-Clashes lists symmetry related clashes.

Mol	Chain	Non-H	H(model)	H(added)	Clashes	Symm-Clashes
1	A	1001	0	875	1	0
2	B	317	0	188	0	0
3	C	333	0	178	0	0
4	A	4	0	0	0	0
5	C	12	0	16	0	0
6	A	46	0	0	0	0
6	B	17	0	0	0	0
6	C	16	0	0	0	0
All	All	1746	0	1257	1	0

The all-atom clashscore is defined as the number of clashes found per 1000 atoms (including

CONFIDENTIAL VALIDATION REPORT

hydrogen atoms). The all-atom clashscore for this structure is 0.

All (1) close contacts within the same asymmetric unit are listed below, sorted by their clash magnitude.

Atom-1	Atom-2	Interatomic distance (Å)	Clash overlap (Å)
1:A:1245:CYS:SG	1:A:1246:ASN:N	2.95	0.40

There are no symmetry-related clashes.

5.3 Torsion angles (i)

5.3.1 Protein backbone (i)

In the following table, the Percentiles column shows the percent Ramachandran outliers of the chain as a percentile score with respect to all X-ray entries followed by that with respect to entries of similar resolution.

The Analysed column shows the number of residues for which the backbone conformation was analysed, and the total number of residues.

Mol	Chain	Analysed	Favoured	Allowed	Outliers	Percentiles	
1	A	129/140 (92%)	114 (88%)	13 (10%)	2 (2%)	11	27

All (2) Ramachandran outliers are listed below:

Mol	Chain	Res	Type
1	A	1240	CYS
1	A	1237	GLU

5.3.2 Protein sidechains (i)

In the following table, the Percentiles column shows the percent sidechain outliers of the chain as a percentile score with respect to all X-ray entries followed by that with respect to entries of similar resolution.

The Analysed column shows the number of residues for which the sidechain conformation was analysed, and the total number of residues.

Mol	Chain	Analysed	Rotameric	Outliers	Percentiles	
1	A	95/127 (75%)	89 (94%)	6 (6%)	20	44

All (6) residues with a non-rotameric sidechain are listed below:

CONFIDENTIAL VALIDATION REPORT

Mol	Chain	Res	Type
1	A	1229	GLU
1	A	1235	ASN
1	A	1246	ASN
1	A	1283	LEU
1	A	1296	LEU
1	A	1304	LYS

Some sidechains can be flipped to improve hydrogen bonding and reduce clashes. There are no such sidechains identified.

5.3.3 RNA (i)

There are no RNA molecules in this entry.

5.4 Non-standard residues in protein, DNA, RNA chains (i)

There are no non-standard protein/DNA/RNA residues in this entry.

5.5 Carbohydrates (i)

There are no carbohydrates in this entry.

5.6 Ligand geometry (i)

Of 6 ligands modelled in this entry, 4 are monoatomic - leaving 2 for Mogul analysis.

In the following table, the Counts columns list the number of bonds (or angles) for which Mogul statistics could be retrieved, the number of bonds (or angles) that are observed in the model and the number of bonds (or angles) that are defined in the Chemical Component Dictionary. The Link column lists molecule types, if any, to which the group is linked. The Z score for a bond length (or angle) is the number of standard deviations the observed value is removed from the expected value. A bond length (or angle) with $|Z| > 2$ is considered an outlier worth inspection. RMSZ is the root-mean-square of all Z scores of the bond lengths (or angles).

Mol	Type	Chain	Res	Link	Bond lengths			Bond angles		
					Counts	RMSZ	$\#\|Z\| > 2$	Counts	RMSZ	$\#\|Z\| > 2$
5	GOL	C	101	-	5,5,5	0.39	0	5,5,5	0.21	0
5	GOL	C	102	-	5,5,5	0.40	0	5,5,5	0.35	0

In the following table, the Chirals column lists the number of chiral outliers, the number of chiral centers analysed, the number of these observed in the model and the number defined in the

CONFIDENTIAL VALIDATION REPORT

Chemical Component Dictionary. Similar counts are reported in the Torsion and Rings columns. '-' means no outliers of that kind were identified.

Mol	Type	Chain	Res	Link	Chirals	Torsions	Rings
5	GOL	C	101	-	-	0/4/4/4	0/0/0/0
5	GOL	C	102	-	-	0/4/4/4	0/0/0/0

There are no bond length outliers.

There are no bond angle outliers.

There are no chirality outliers.

There are no torsion outliers.

There are no ring outliers.

No monomer is involved in short contacts.

5.7 Other polymers (i)

There are no such residues in this entry.

5.8 Polymer linkage issues (i)

There are no chain breaks in this entry.

CONFIDENTIAL VALIDATION REPORT

6 Fit of model and data (i)

6.1 Protein, DNA and RNA chains (i)

In the following table, the column labelled '#RSRZ> 2' contains the number (and percentage) of RSRZ outliers, followed by percent RSRZ outliers for the chain as percentile scores relative to all X-ray entries and entries of similar resolution. The OWAB column contains the minimum, median, 95th percentile and maximum values of the occupancy-weighted average B-factor per residue. The column labelled 'Q< 0.9' lists the number of (and percentage) of residues with an average occupancy less than 0.9.

Mol	Chain	Analysed	<RSRZ>	#RSRZ>2			OWAB(Å^2)	Q<0.9
1	A	131/140 (93%)	1.26	25 (19%)	1	1	25, 48, 139, 154	0
2	B	16/16 (100%)	-0.28	0	100	100	23, 38, 53, 61	0
3	C	16/16 (100%)	-0.31	0	100	100	29, 39, 59, 63	0
All	All	163/172 (94%)	0.96	25 (15%)	2	1	23, 45, 137, 154	0

All (25) RSRZ outliers are listed below:

Mol	Chain	Res	Type	RSRZ
1	A	1227	GLU	11.3
1	A	1237	GLU	9.4
1	A	1236	GLU	8.2
1	A	1241	ALA	7.9
1	A	1233	GLU	7.8
1	A	1240	CYS	7.7
1	A	1234	GLU	6.8
1	A	1228	GLU	6.8
1	A	1229	GLU	6.7
1	A	1226	LYS	6.4
1	A	1239	GLU	6.2
1	A	1225	GLU	5.8
1	A	1235	ASN	5.5
1	A	1231	GLU	5.4
1	A	1230	GLU	5.0
1	A	1238	GLU	4.5
1	A	1232	GLU	4.2
1	A	1278	PHE	3.8
1	A	1252	MET	3.2
1	A	1247	MET	3.0
1	A	1242	ALA	2.9
1	A	1243	TYR	2.3
1	A	1262	LEU	2.3

Continued on next page...

CONFIDENTIAL VALIDATION REPORT

Continued from previous page...

Mol	Chain	Res	Type	RSRZ
1	A	1254	PHE	2.2
1	A	1251	THR	2.0

6.2 Non-standard residues in protein, DNA, RNA chains (i)

There are no non-standard protein/DNA/RNA residues in this entry.

6.3 Carbohydrates (i)

There are no carbohydrates in this entry.

6.4 Ligands (i)

In the following table, the Atoms column lists the number of modelled atoms in the group and the number defined in the chemical component dictionary. The B-factors column lists the minimum, median, 95th percentile and maximum values of B factors of atoms in the group. The column labelled 'Q< 0.9' lists the number of atoms with occupancy less than 0.9.

Mol	Type	Chain	Res	Atoms	RSCC	RSR	B-factors(Å^2)	Q<0.9
5	GOL	C	102	6/6	0.65	0.26	52,54,56,56	0
5	GOL	C	101	6/6	0.89	0.26	33,34,35,35	0
4	ZN	A	1401	1/1	0.97	0.08	60,60,60,60	1
4	ZN	A	1404	1/1	0.99	0.11	34,34,34,34	0
4	ZN	A	1402	1/1	1.00	0.15	38,38,38,38	0
4	ZN	A	1403	1/1	1.00	0.12	33,33,33,33	0

6.5 Other polymers (i)

There are no such residues in this entry.

CONFIDENTIAL VALIDATION REPORT
